# Efficient quantum algorithms for set operations

**DOI:** 10.1038/s41598-024-56860-2

**Published:** 2024-03-25

**Authors:** Rehab Elgendy, Ahmed Younes, H. M. Abu-Donia, R. M. Farouk

**Affiliations:** 1 Department of Mathematics, Faculty of Science, Zagazig University, Zagazig, Egypt; 2 Department of Mathematics and Computer Science, Faculty of Science, Alexandria University, Alexandria, Egypt; 3 School of Computer Science, University of Birmingham, Birmingham, B15 2TT UK

**Keywords:** Set operations, Quantum search algorithm, Union, Intersection, Difference, Amplitude amplification, Incomplete superposition, Mathematics and computing, Computer science, Physics, Quantum physics, Quantum information, Quantum simulation, Qubits, Mathematics and computing, Computer science, Physics, Quantum physics, Quantum information, Quantum simulation, Qubits

## Abstract

Analyzing the relations between Boolean functions has many applications in many fields, such as database systems, cryptography, and collision problems. This paper proposes four quantum algorithms that use amplitude amplification techniques to perform set operations, including Intersection, Difference, and Union, on two Boolean functions in $\mathcal {O}(\sqrt{\mathcal {N}} )$ time complexity. The proposed algorithms employ two quantum amplitude amplification techniques divided into two stages. The first stage uses the Younes et al. algorithm for quantum searching via entanglement and partial diffusion to prepare incomplete superpositions of the truth set of the first Boolean function. In the second stage, a modified version of Arima’s algorithm, along with an oracle that represent the second Boolean function, is employed to handle the set operations. The proposed algorithms have a higher probability of success in more general and comprehensive applications when compared with relevant techniques in literature.

## Introduction

Quantum computers^1–3^ are probabilistic devices that can speed up computations compared to classical computers^4,5^. Many quantum algorithms have been presented^6–12^. For instance, Shor’s algorithm^10^ is a polynomial-time algorithm to obtain the prime factors of an integer *n*. Grover^6^ presented a quantum search algorithm to look for an item among an unstructured list of $\mathcal {N}$ items with a quadratic speedup compared to classical algorithms. Grover’s algorithm has motivated researchers to analyze and; or generalize his algorithm^13–18^. Grover’s search algorithm is optimal in the case of a single match in the search space; increasing iterations of Grover’s algorithm makes the problem more complicated^19^. Grover’s algorithm is effective when the distribution of the dataset in the initial amplitude is uniform^20–22^. Another quantum search algorithm is Ventura’s algorithm^22^, which generalizes the Grover algorithm and is effective when the distribution of the dataset in the initial amplitude is not uniform^20^. Arima’s search algorithm^21^ also generalizes the Grover algorithm and improves Ventura’s algorithm’s performance.

Set operations are crucial in data analysis and organization when handling sets. There are four operations: intersection, difference, union, and complement. The intersection operation identifies shared elements among sets, while the difference operation extracts elements from one set compared to another. The union operation combines elements from sets without repetition. The complement represents all elements not included in a specific set from the universal set. Set operations find use in fields such as data analysis for merging datasets, pinpointing commonalities, and removing redundant information. In computer science, they are vital for algorithms related to searching, sorting, and optimizing data structures. Additionally, in databases and information retrieval systems, set operations support querying and manipulation of datasets^23^. Moreover, set operations are utilized in intelligence tasks such as training networks and optimizing machine learning models^24,25^. They are also used in algorithms related to graph theory and networking^26^. By applying principles from quantum computing, set operations can be carried out with increased efficiency and computational power compared to other methods. Quantum algorithms, like those outlined in this paper, offer benefits over conventional set operations. These quantum algorithms make use of amplitude amplification techniques that allow for the completion of set operations such as intersection, Difference and Union in $\mathcal {O}(\sqrt{\mathcal {N}} )$. This marks an enhancement in efficiency when compared to related algorithms^27–29^. Moreover, the incorporation of quantum search algorithms such as Younes et al.’s algorithm and a modified version of Arima’s algorithm makes it possible to leverage quantum entanglement and partial diffusion, resulting in higher success rates and broader applications. These advancements in quantum algorithms for set operations provides an advancement compared to traditional methods, paving the way for new opportunities in database systems, cryptography, and collision problem domains^24,25,30,31^.

In 2000, Heiligman presented an algorithm that requires $\mathcal {O}(\mathcal {N}^\frac{3}{4}\log \mathcal {N})$ to find matches between two databases^32^. In 2003, Younes et al. presented a quantum algorithm^18^ to find multiple solutions to the oracle $U_f$ in $\mathcal {O}\left( \sqrt{ \mathcal {N}/ \mathcal {C}}\right)$, where $\mathcal {C}$ is the number of common entries. In 2005, P. Mateus presented a quantum algorithm^33^ for closest pattern matching of size *m* in $\mathcal {O}\left( \sqrt{ \mathcal {S}}\right)$ where $\mathcal {S}$ is the size of the string. In 2012, A. Tulsi presented a quantum algorithm^34^ to find a single common element between two sets in $\mathcal {O}\left( \sqrt{\mathcal {N}}\right)$. In 2013, Pang et al. presented a quantum algorithm^27^ for set operations. Pang et al. found common intersected elements between two sets *A* and *B* using a similar algorithm proposed in this paper^15^ in $\mathcal {O}\left( \left| A\right| \times \left| B\right| \times \left| I\right| \right)$ for set operation $I=A\cap B$. In 2017, K. El-Wazan presented an algorithm^28^ that solves the problem: Given $\mathcal {L}$ databases of unstructured entries, find the common entries $\mathcal {C}$ between those databases in $\mathcal {O}\left( \mathcal {L}\sqrt{\mathcal {M}}\right)$, where $\mathcal {M}$ is the number of records for each database. K. El-Wazan’s algorithm proved that when the given $\mathcal {L}$ databases are of the same size, it will require $\mathcal {O}\left( \mathcal {L}\sqrt{\mathcal {M}/{\mathcal {C}}}\right)$ oracle calls. Moreover, the performance of K. El-Wazan’s algorithm is more reliable in the case of multiple matches. In 2020, A. Kiss and K. Varga presented an algorithm^35^ to combine two oracles and determine how similar they are using the Deutsch–Jozsa algorithm. In this case, being similar means that the sets of marked states have just few ones outside their intersection. In 2020, S. Jóczik and A. Kiss presented an algorithm^29^ to find the set operations between two sets in $\mathcal {O}\left( \sqrt{\mathcal {N}}\right)$ using Grover’s algorithm.

The aim of the paper is to propose quantum algorithms to find the Intersection, Difference and Union between any two Boolean functions. The proposed algorithms consist of two stages: the first stage uses an oracle that represents the first Boolean function to prepare an incomplete superposition of a search space of the truth set of the first Boolean function using the Younes et al. algorithm for quantum searching via entanglement^36^. The second stage uses an oracle that represents the second Boolean function to search for a solution that represents the result of the set operator in the prepared incomplete superposition using an updated version of Arima’s algorithm for incomplete superposition searching^21^. This paper presents four new quantum algorithms designed to carry out set operations on functions. Specifically, the paper outlines the following algorithms:

1. Two quantum algorithms are introduced that effectively identify both True and False intersections between two Boolean functions.
2. Another quantum algorithm is detailed for computing the difference between two Boolean functions.
3. Additionally, a quantum algorithm is described for determining the union between two Boolean functions.

The paper’s organization is as follows: Section 2 includes the required background and the quantum search algorithms applied in the proposed algorithms. Section 3 proposes the quantum algorithms to find the intersection, the difference and the union between two Boolean functions and applies an example to each proposed algorithm. Section 4 analysis of the searching phase. Section 5 discusses the proposed algorithm. Section 6 concludes the paper and mentions future work.

## Background

### Set operations on Boolean functions

A Boolean function *f* is a function whose variables (arguments) take the values 0 (False) or 1 (True), i.e., *f* can be represented as follows $f: Y^n\rightarrow Y$, such that $Y\ =\{0,\ 1\}$. The domain $Y^n$ of *f* is the set of ${2}^{n}$ binary vectors.

Let $f^F\subseteq Y^n$ be the set of binary vectors where the Boolean function *f* evaluated to False and $f^T\subseteq Y^n$ be the set of binary vectors where the Boolean function *f* evaluated to True, where $f^T\cup f^F=Y^n$.

Let $f_1$ and $f_2$ be two Boolean functions; we define the set operations over Boolean functions as follows:

1. Intersection: $f_1\cap f_2$ to be the set of binary vectors that evaluates both $f_1$ and $f_2$ to True at the same time, i. e. $f_1\cap f_2=f^ T_1\cap f_2^T$.
2. Union: $f_1\cup f_2$ to be the set of binary vectors that evaluates either $f_1$ or $f_2$ to True, i.e. $f_1\cup f_2= f_1^T\cup f_2^T$.
3. Difference: $f_1-f_2$ to be the set of binary vectors that evaluates $f_1$ to True and evaluates $f_2$ to False, i. e. $f_1-f_2= f_1^T-f_2^T$ such that $f_1-f_2\ne f_2-f_1$.

#### Example

Let $n = 4$ then $Y^n =\{0, 1, 2, 3, 4, 5, 6, 7, 8, 9,10,11,12,13,14,15\}$, where the integers in $Y^n$ represent the integer representation of the binary vectors. Given two Boolean functions $f_1$ and $f_2$ such that $f_1^T=\{0,1,3,5,7,11,15\}$ and $f_2^T=\{0,2,3,6,7,8,15\}$ then $f_1\cap f_2=\{0,3,7,15\}$, $f_1\cup f_2=\{0,1,2,3,5,6,7,8,11,15\}$, $f_1-f_2=\{1,5,11\}$ and $f_2-f_1=\{2,6,8\}$.

### Quantum computing

The quantum bit or qubit^37^ is the elementary unit of the data in quantum computing. The qubit can be in a combination of $\left. |0\right\rangle$ and $|\left. 1\right\rangle$ in a linear superposition as shown in Eq. 1, where $\alpha$, $\beta$ are complex numbers representing the probabilistic amplitudes of $\left. |0\right\rangle$ and $\left. |1\right\rangle$ respectively, and the condition in Eq. 2 must be satisfied by the amplitudes^38^,

1 $$\begin{aligned} \left. |\psi \right\rangle =\alpha \left. |0\right\rangle +\beta \left. |1\right\rangle , \end{aligned}$$

and

2 $$\begin{aligned} |\alpha |^2+|\beta |^2=1, \end{aligned}$$

where $\left| \alpha \right| ^2=\alpha .\alpha ^*, \alpha ^*$ is the complex conjugate transpose of $\alpha$. When a measurement is carried out, the superposition is collapsed to one of the states in a probabilistic way, i. e., the superposition is collapsed to $\left. |0\right\rangle$ with probability $\left| \alpha \right| ^2$ and $\left. |1\right\rangle$ with probability $|\beta |^2$.

Entanglement is one of the quantum features in which the quantum state has to be described for the whole system^39^, and each object of the quantum system cannot be described independently. Another property of quantum computing is parallelism, where it takes a quantum computer a single step to operate on *n* inputs with a single gate. In contrast, the classical computer takes $2^n$ steps for the same input size. Parallelism performs multiple operations at a time and does not require additional hardware or wait for other processes to complete. Quantum gates are unitary operators^19^, supposing that a gate has *n* inputs, and then it can be represented as $2^n\times 2^n$ unitary matrix assuming that state $$\left. |0\right\rangle =\left[ \begin{array}{c} 1 \\ 0 \end{array} \right]$$ and state$$\left. |1\right\rangle =\left[ \begin{array}{c} 0 \\ 1 \end{array} \right]$$.

    Some of the quantum gates^40^ which will be used in the paper are:

The *X* gate is similar to the NOT gate in classical computers, where it maps $\left. |0\right\rangle$ to $\left. |1\right\rangle$ and $\left. |1\right\rangle$ to $\left. |0\right\rangle$ as shown in the following equation:

3 $$\begin{aligned} X. \left. |0\right\rangle= & {} \left[ \begin{array}{cc} 0 &{} \ 1 \\ 1 &{} \ 0 \end{array} \right] . \left[ \begin{array}{c} 1 \\ 0 \end{array} \right] =\left[ \begin{array}{c} 0 \\ 1 \end{array} \right] =\left. |1\right\rangle , \end{aligned}$$

4 $$\begin{aligned} X. \left. |1\right\rangle= & {} \left[ \begin{array}{cc} 0 &{} \ 1 \\ 1 &{} \ 0 \end{array} \right] . \left[ \begin{array}{c} 0 \\ 1 \end{array} \right] =\left[ \begin{array}{c} 1 \\ 0 \end{array} \right] =\left. |0\right\rangle . \end{aligned}$$

The Hadamard gate has the following effect when applied on $\left. |0\right\rangle$ and $\left. |1\right\rangle$:

5 $$\begin{aligned} H. \left. |0\right\rangle= & {} \frac{1}{\sqrt{2}}\left[ \begin{array}{cc} 1 &{} \ \ 1 \\ 1 &{} -1 \end{array} \right] . \left[ \begin{array}{c} 1 \\ 0 \end{array} \right] =\frac{1}{\sqrt{2}}\left[ \begin{array}{c} 1 \\ 1 \end{array} \right] =\frac{1}{\sqrt{2}}\left( \left. |0\right\rangle +\left. |1\right\rangle \right) , \end{aligned}$$

6 $$\begin{aligned} H. \left. |1\right\rangle= & {} \frac{1}{\sqrt{2}}\left[ \begin{array}{cc} 1 &{} \ \ 1 \\ 1 &{} -1 \end{array} \right] . \left[ \begin{array}{c} 0 \\ 1 \end{array} \right] =\frac{1}{\sqrt{2}}\left[ \begin{array}{c} \ \ 1 \\ -1 \end{array} \right] =\frac{1}{\sqrt{2}}\left( \left. |0\right\rangle -\left. |1\right\rangle \right) . \end{aligned}$$

The *Z* gate that does not vary the state$\left. |0\right\rangle$ but it converts $\left. |1\right\rangle$ to $-\left. |1\right\rangle$ as shown in the following

7 $$\begin{aligned} Z. \left. |0\right\rangle= & {} \left[ \begin{array}{cc} 1 &{} \ \ \ 0 \\ 0 &{} -1 \end{array} \right] . \left[ \begin{array}{c} 1 \\ 0 \end{array} \right] =\left[ \begin{array}{c} 1 \\ 0 \end{array} \right] =\left. |0\right\rangle , \end{aligned}$$

8 $$\begin{aligned} Z. \left. |1\right\rangle= & {} \left[ \begin{array}{cc} 1 &{} \ \ 0 \\ 0 &{} -1 \end{array} \right] . \left[ \begin{array}{c} 0 \\ 1 \end{array} \right] =\left[ \begin{array}{c} \ \ 0 \\ -1 \end{array} \right] =-\left. |1\right\rangle . \end{aligned}$$

Quantum circuits are a cascade of basic quantum gates to carry out a specific operation.

### Quantum searching algorithms

This section reviews the four quantum algorithms that will be used in the proposed algorithm: Grover’s algorithm for searching an unstructured database uses Grover’s diffusion operator *G* (inversion about the mean)^36^. Younes et al. algorithm for searching an unstructured list using a quantum operator $D_p$ that performs the inversion about the mean only on a subspace of the system (Partial Diffusion Operator)^36^. Ventura’s algorithm searches in an incomplete superposition when the initial amplitude distribution of the dataset is non-uniform, and Arima’s algorithm improved Ventura’s algorithm to increase the probability of results^21^.

#### Grover’s algorithm

Grover presented a quantum algorithm to search an unstructured database of ${\mathcal {N}}$ items in $\mathcal {O}\left( \sqrt{\mathcal {N}}\right)$. Grover’s algorithm prepares a quantum register with $n + 1$ qubits in a uniform superposition of qubits where the first *n* qubits are initialized to the state $\left. |0\right\rangle$ and an extra workspace qubit initialized to the state $\left. |1\right\rangle$^36^,

9 $$\begin{aligned} \left. |{\psi }_{G0}\right\rangle ={\left. |0\right\rangle }^{\otimes n}\ \otimes \ \left. |1\right\rangle , \end{aligned}$$

Then, the following steps must be iterated approximately $\left\lfloor \frac{\pi \sqrt{{\mathcal {N}}}}{4}\right\rfloor$ times, where $\lfloor \rfloor$ is the floor operation.

Grover’s algorithm applies Hadamard gates on the $n+1$ qubits as follows:

10 $$\begin{aligned} \left. |{\psi }_{G1}\right\rangle =\frac{1}{\sqrt{\mathcal N}}\sum ^{{\mathcal {N}}-1}_{r=0}{\left. |r\right\rangle \otimes \left[ \frac{\left. |0\right\rangle -\left. |1\right\rangle }{\sqrt{2}}\right] .} \end{aligned}$$

After that, it applies the oracle operator $O_G$ that provides the amplitudes of the matches phase shift of $e^{i\pi }$^36^ and evaluates a Boolean function $f_G:\ \{0,\ 1{\}}^n\rightarrow \{0,\ 1\}$ as shown in Eq. 11.

11 $$\begin{aligned} O_G\left. |x\right\rangle =\left\{ \begin{array}{c} \ \ \ \left. |x\right\rangle ,\ \ \ \ f_G\left( x\right) =0 \\ -\left. |x\right\rangle ,\ \ \ \ f_G\left( x\right) =1 \end{array} \right. \ \end{aligned}$$

The algorithm then applies the diffusion operator *G* on the first *n* qubits to make inversion about the mean, and *G* is shown in Eq. 12 :

12 $$\begin{aligned} G=H^{\otimes n}\left( 2{\left. |0\right\rangle }^{\otimes n}{\left\langle 0|\right. }^{\otimes n}-I_n\right) H^{\otimes n}, \end{aligned}$$

where $I_n$ is the identity matrix of size ${\mathcal {N}}\times {\mathcal {N}}$ . Measurement is then applied on the first *n* qubits to retrieve one of the searched items. Assume that $\sum {'}$ is the sum over the desired ${\mathcal {C}}$ matches, while $\sum {''}$ represents the sum over the ${\mathcal {N}}-{\mathcal {C}}$ undesired matches. The system after $q_G\ \ge \ 1$ iterations can be written as follows,

13 $$\begin{aligned} \left. |\psi _G\right\rangle =b_g\sum ^{\mathcal N-1}_{r=0}{''\left. |r\right\rangle }+a_g\sum ^{\mathcal N-1}_{r=0}{'\left. |r\right\rangle }, \end{aligned}$$

where the amplitudes $a_g$ and $b_g$ after $q_G\ge \ 1$ iterations are defined by the following recurrence relations^15^.

14 $$\begin{aligned} a_0= & {} b_0=\frac{1}{\sqrt{{\mathcal {N}}}},\nonumber \\ a_g= & {} \frac{{\mathcal {N}}-2{\mathcal {C}}}{N}a_{g-1}+\frac{2\left( {\mathcal {N}}-{\mathcal {C}}\right) }{{\mathcal {N}}}b_{g-1},\nonumber \\ b_g= & {} \frac{{\mathcal {N}}-2{\mathcal {C}}}{{\mathcal {N}}}b_{g-1}+\frac{2\left( {\mathcal {N}}-{\mathcal {C}}\right) }{{\mathcal {N}}}a_{g-1}. \end{aligned}$$

Solving these recurrence relations, the closed forms can be written as follows^15^:

15 $$\begin{aligned} a_g=\frac{1}{\sqrt{{\mathcal {C}}}}{\sin \left( \left( 2q_G+1\right) \theta \right) \ },\ b_g=\frac{1}{\sqrt{{\mathcal {N}}-{\mathcal {C}}}}{\cos \left( \left( 2q_G+1\right) \theta \right) \ }, \end{aligned}$$

where $${sin}^2(\theta )={{\mathcal {C}}}/{{\mathcal {N}}\ \ ,\ 0\le \theta \le {\pi }/{2}},\ q_G=\frac{\pi }{4}\sqrt{\frac{{\mathcal {N}}}{{\mathcal {C}}}}$$

#### Younes et al. algorithm

Younes *et al.* algorithm can search an unstructured database of ${\mathcal {N}}$ items with a higher probability of success via the partial diffusion operator $\left( D_P\right)$ in $\mathcal {O}\left( \sqrt{{\mathcal {N}}/\mathcal C}\right)$ where ${\mathcal {C}}$ is the number of matches satisfying $1\le {\mathcal {C}}\le {\mathcal {N}}$. This algorithm prepares a complete superposition and amplifies the solutions’ amplitudes via entanglement of the search space with the extra qubit, which is useful in that it can be done by applying the measurement on the extra qubit^18,36^.

This algorithm applies the oracle operator $O_Y$ on $n+1$ qubits where $O_Y$ evaluates the Boolean function $f_Y:\ \{0,\ 1{\}}^n\rightarrow \{0,\ 1\}$ as shown in the following equation:

16 $$\begin{aligned} O_Y\left. |x,\ 0\right\rangle =\left\{ \begin{array}{c} \left. |x,\ 0\right\rangle ,\ \ \ \ f_Y\left( x\right) =0 \\ \left. |x,\ 1\right\rangle ,\ \ \ \ f_Y\left( x\right) =1 \end{array} \right. \end{aligned}$$

Then, the algorithm applies the $D_P$ the operator, which can take the form as described in the following equation^36^:

17 $$\begin{aligned} D_P=\left( H^{\otimes n}\otimes {\ I}_1\right) \left( 2{\left. |0\right\rangle }^{\otimes n+1}{\left\langle 0|\right. }^{\otimes n+1}-I_{n+1}\right) \left( H^{\otimes n}\otimes {\ I}_1\right) , \end{aligned}$$

where $\left. |0\right\rangle$’s size is $2\mathcal N=2^{n+1}$, and the identity matrix $I_k$ is of size $2^k\times 2^k$.

Suppose a general system $\left. |{\psi }_{Y2}\right\rangle$ of $n+1$ qubits as follows:

18 $$\begin{aligned} \left. |{\psi }_{Y2}\right\rangle =\sum ^{\mathcal N-1}_{r=0}{\left. {\alpha }_r|r\right\rangle \otimes \left. |0\right\rangle +\sum ^{{\mathcal {N}}-1}_{r=0}{\left. {\beta }_r|r\right\rangle \otimes \left. |1\right\rangle ,}} \end{aligned}$$

where ${\alpha }_r={\delta }_k:k\ even$ and ${\beta }_r={\delta }_k:k$* odd*.

Hence, applying $D_P$ on the general system has the following effect:

19 $$\begin{aligned} D_P\left. |{\psi }_{Y2}\right\rangle =\sum ^{\mathcal N-1}_{r=0}{(2\left\langle \alpha \right\rangle -{\alpha }_r)\left( \left. |r\right\rangle \otimes \left. |0\right\rangle \right) }-\sum ^{{\mathcal {N}}-1}_{r=0}{\left. {\beta }_r|r\right\rangle \otimes \left. |1\right\rangle ,} \end{aligned}$$

where $\left\langle \alpha \right\rangle =\frac{1}{\sqrt{{\mathcal {N}}}}\sum ^{{\mathcal {N}}-1}_{r=0}{{\alpha }_r/{\mathcal {N}}}$ is the mean of the amplitudes of the subspace entangled with the $\left. |0\right\rangle$ of the extra qubit. The $O_Y$ and $D_P$ operators are iterated $q_y$ times where $q_y$ is as follows:

20 $$\begin{aligned} q_y=\left\lfloor \frac{\pi }{2\sqrt{2}}\sqrt{\frac{\mathcal N}{{\mathcal {C}}}}\right\rfloor , 1\le {\mathcal {C}}\le {\mathcal {N}}. \end{aligned}$$

Assume that $\sum {'}$ is the sum over the desired ${\mathcal {C}}$ matches, while $\sum {''}$ represents the sum over the ${\mathcal {N}}-{\mathcal {C}}$ undesired matches. The system after $q_y>1$ iterations can be described as follows:

21 $$\begin{aligned} \left. |{\psi }_{Y2}\right\rangle =a_q\sum ^{\mathcal N-1}_{r=0}{''(\left. |r\right\rangle \otimes \left. |0\right\rangle )+b_q\sum ^{{\mathcal {N}}-1}_{r=0}{'(\left. |r\right\rangle \otimes \left. |0\right\rangle )+c_q\sum ^{\mathcal N-1}_{r=0}{'(\left. |r\right\rangle \otimes \left. |1\right\rangle )}.}} \end{aligned}$$

Finally, the first *n *qubits are measured to obtain one of the searched items.

#### Ventura’s algorithm

It is known that Grover’s algorithm is effective in the case where the initial amplitude distribution of the dataset is uniform but is not always effective in the non-uniform case. Therefore, the Ventura algorithm was proposed to solve the search in an incomplete superposition when the initial amplitude distribution of the dataset is non-uniform.

Suppose $\left. |\psi \right\rangle$ is an incomplete superposition, and Let $I_{ f_1^T}$ and $I_{ f_2^T}$ be phase oracles that mark two sets of *n*-qubit states $f_1^T$ and $f_2^T$ with $|f_{1}^{T}|, |f_{2}^{T}|<< {\mathcal {N}}$ where $I_{ f_1^T}$ marks the searching states and $I_{ f_2^T}$ marks any state in stored data *m*. Ventura’s algorithm can be summarized as follows^22^.

Given: Phase oracles $I_{ f_1^T}$* and *$I_{ f_2^T}$

1. Denote $${\mathcal {R}}_{ f_1^T}={G I}_{ f_1^T}$$ and $${\mathcal {R}}_{ f_2^T}=G I_{ f_2^T}$$ .
2. Suppose $$\left. |\psi \right\rangle =\frac{1}{m}\sum ^m_{r=1}{\left. |r\right\rangle }$$.
3. $$\left. |\psi { ' }\right\rangle = \mathcal {R}_{ f_2^T} \left. |\psi \right\rangle$$.
4. $$\left. |\psi { '' }\right\rangle = {\mathcal {R}}_{ f_1^T} \left. |\psi { ' }\right\rangle$$.
5. Repeat $$t=\left\lfloor {\pi }/{4}\sqrt{{\mathcal {N}}}-2\right\rfloor$$ times.
6. $$\left. |\psi { ''' }\right\rangle = {\mathcal {R}}_{ f_2^T}\left. |\psi {''}\right\rangle$$.
7. Observe the system.

#### Arima’s algorithm

Arima search algorithm was proposed to solve the search in an incomplete superposition when the initial amplitude distribution of the dataset in non-uniform cases means that ${\mathcal {N}}$ does not equal the number of stored data and improves the venture search performance algorithm. Arima’s algorithm can be summarized as follows^21^,

Given: Phase oracles $I_{ f_1^T}$* and *$I_{ f_2^T}$

1. Denote $${\mathcal {R}}_{ f_1^T}={G I}_{ f_1^T}$$ and $${\mathcal {R}}_{ f_2^T}=GI_{ f_2^T}$$ .
2. Suppose $$\left. |\psi \right\rangle =\frac{1}{m}\sum ^m_{r=1}{\left. |r\right\rangle }$$.
3. Repeat $$P =\left\lfloor {(\pi \sqrt{2{\mathcal {N}}}) }/{8}\right\rfloor$$ times.
4. $$\left. |\psi {'} \right\rangle = {\mathcal {R}}_{ f_2^T}\left. |\psi \right\rangle$$.
5. $$\left. |\psi {''} \right\rangle ={\mathcal {R}}_{ f_1^T}\left. |\psi {'}\right\rangle$$.
6. Observe the system.

## The proposed algorithms

In this section, we will propose algorithms supposing that $f_1$ and $f_2$ are two Boolean functions with *n* Boolean inputs and $f_1^T$ and $f_2^T$ are the set of binary vectors where the Boolean function $f_1$ and $f_2$ evaluated to True respectively. The proposed algorithms consist of two stages: the first stage prepares an incomplete superposition of a search space with specific properties using the Younes et al. algorithm^36^ for searching a state that satisfies the oracle that represents the $I_{ f_1^T}$. The second stage prepares an incomplete superposition of a search space with specific properties using an updated version of Arima’s algorithm^21,41^ for searching a state in the oracle $I_{ f_2^T}$ that satisfies with a state in the oracle ${ I}_{ f_1^T}$.

### The proposed quantum algorithm for true intersection operation

Given two Boolean functions $f_1$ and $f_2$, it is required to find the set of binary vectors that evaluates both $f_1$ and $f_2$ to True simultaneously, i. e., to find the intersection between them, the steps of the proposed algorithm will be illustrated using this example: If $n=4$, the possible number of items to the Boolean function equals ${\mathcal {N}}$, where ${\mathcal {N}}=16$. Assume that the number of stored elements in $f_{{ 1}}$ and $f_{{ 2}}$ equals *m*, where $m=8$. Suppose that $f_{{ 1}}$ evaluates to true for each pattern in the set $\{\left. { |0}\right\rangle { ,}\left. { |1}\right\rangle { ,}\left. { |3}\right\rangle { ,}\left. { |5}\right\rangle { ,}\left. { |7}\right\rangle { ,}\left. { |9}\right\rangle { ,}\left. { |11}\right\rangle { ,}\left. { |15}\right\rangle \},$ and $f_{{ 2}}$ evaluates to true for each pattern in the set {$\left. { |0}\right\rangle ,\left. { |2}\right\rangle {,}\left. { |4}\right\rangle { ,}\left. { |6}\right\rangle { ,}\left. { |8}\right\rangle { ,}\left. { |10}\right\rangle { ,}\left. {|12}\right\rangle { ,}\left. {|15}\right\rangle$}.

Apply Younes et al. algorithm^36^ as follows:
  - The preparation of the register consists of $$n+1$$ qubits, and all of them are in the state $$\left. |0\right\rangle$$. The auxiliary qubit is used to evaluate the Boolean function $$f_1$$.
    22 $$\begin{aligned} \left. \left| \ {\psi }_0\right. \right\rangle =\left. \left| 0\right. \right\rangle ^{\otimes n} \otimes \left. \left| 0\right. \right\rangle . \end{aligned}$$
    When applying Eq. (22) to the illustrative example, the form will be as follows:
    23 $$\begin{aligned} \left. \left| {{\psi }}_0\right. \right\rangle ={\left. |0\right\rangle }^{\otimes 4}\otimes \left. |0\right\rangle =\left. |00000\right\rangle . \end{aligned}$$
  - The initialization of the register in which the Hadamard gate *H* is applied on the first *n* qubits in parallel as in Eq. (24).
    24 $$\begin{aligned} \left. \left| \psi _1\right. \right\rangle \ {}&=\ ({H}^{\otimes n}\otimes \ I) \left. \left| \psi _0\right. \right\rangle .\nonumber \\&=\frac{1}{\sqrt{2^n}}\ \sum _{r=0}^{2^n-1}{\ \left. \left| r\right. \right\rangle }\otimes \left. \left| 0\right. \right\rangle \nonumber \\&= \frac{1}{\sqrt{2^n}}\sum _{ r\in {[0,1]^{n}}} \left. \left| r, 0\right. \right\rangle . \end{aligned}$$
    When applying Eq. (24) to the illustrative example, the form will be as follows:
    25 $$\begin{aligned} \left. |\psi _1\right\rangle&=\left( H^{\otimes 4}\otimes I\right) \left. \left| {{\psi }}_0\right. \right\rangle =\left( H^{\otimes 4}\otimes I\right) \left. |00000\right\rangle \nonumber \\&=\frac{1}{4}(\left. |00000\right\rangle +\left. |00010\right\rangle +\left. |00100\right\rangle +\left. |00110\right\rangle +\left. |01000\right\rangle \nonumber \\&\quad +\left. |01010\right\rangle +\left. |01100\right\rangle +\left. |01110\right\rangle +\left. |10000\right\rangle +\left. |10010\right\rangle \nonumber \\&\quad + \left. |10100\right\rangle +\left. |10110\right\rangle +\left. |11000\right\rangle +\left. |11010\right\rangle +\left. |11100\right\rangle \nonumber \\&\quad +\left. |11110\right\rangle ). \end{aligned}$$
  - This algorithm iterates the following steps for $$\left\lfloor \frac{\pi }{2\sqrt{2}}\sqrt{\frac{{\mathcal {N}}}{\mathcal C}}\right\rfloor$$.
    (i) Apply the oracle operator $$I_{f_{1}^{T}}$$ on $$n+1$$ qubits where $$I_{f_{1}^{T}}$$ evaluates the first boolean function $$f_1$$ as follows:
    26 $$\begin{aligned} \left. \left| \psi _2\right. \right\rangle \ {}&=I_{ f_1^T}\left. \left| \psi _1\right. \right\rangle .\nonumber \\&=\frac{1}{\sqrt{2^n}}\ \sum _{r=0}^{2^n-1}{\ \left. \left| r\right. \right\rangle }\otimes \left. \left| f_1(r)\right. \right\rangle ,\nonumber \\&=\frac{1}{\sqrt{2^n}}\sum _{ r\in {[0,1]^{n}}} \left. \left| r, f_1(r)\right. \right\rangle , \end{aligned}$$
    such that
    27 $$\begin{aligned} I_{ f_1^T}|r, 0\rangle =\left\{ \begin{array}{l} |r, 0\rangle ,\ \ \ f_{1}(r)=0. \\ |r, 1\rangle , \ \ \ f_{1}(r)=1. \end{array}\right. \end{aligned}$$
    When applying Eq. (26) to the illustrative example, the form will be as follows:
    28 $$\begin{aligned} \left. |\psi _2\right\rangle&=I_{ f_1^T}\left. |\psi _1\right\rangle =I_{ f_1^T}\left( H^{\otimes 4}\otimes I\right) \left. |00000\right\rangle \nonumber \\&=\frac{1}{4}(\left. |00001\right\rangle +\left. |00011\right\rangle +\left. |00100\right\rangle +\left. |00111\right\rangle +\left. |01000\right\rangle \nonumber \\&\quad +\left. |01011\right\rangle +\left. |01100\right\rangle +\left. |01111\right\rangle +\left. |10000\right\rangle +\left. |10011\right\rangle \nonumber \\&\quad +\left. |10100\right\rangle +\left. |10111\right\rangle +\left. |11000\right\rangle +\left. |11010\right\rangle +\left. |11100\right\rangle \nonumber \\&\quad +\left. |11111\right\rangle ). \end{aligned}$$
    (ii) After that, the algorithm applies the partial diffusion $$D_p$$ on the $$n+1$$ qubits. Assume that $${\mathcal {C}}$$ be the number of elements common to $$f_1^T$$ and $$f_2^T$$ such that $$1\le {\mathcal {C}}\le {\mathcal {N}}$$. Let $$\sum _{r}{'}$$ represents the intersected items and $$\sum _{r}{''}$$ represents undesired items in the truth set so $$\left. \left| \psi _2\right. \right\rangle$$ can be rewritten as follows:
    29 $$\begin{aligned} \left. \left| \psi _2\right. \right\rangle 
=\frac{1}{\sqrt{2^n}}\sum _{r=0}^{2^n-1}{'' \ (}\left. \left| r\right. \right\rangle \otimes \left. \left| 0\right. \right\rangle )+\frac{1}{\sqrt{2^n}}\sum _{r=0}^{2^n-1}{'\ (\left. \left| r\right. \right\rangle \otimes \left. \left| 1\right. \right\rangle ).} \end{aligned}$$
    After applying $$D_p$$ to $$\left. \left| \psi _2\right. \right\rangle$$ the system can be described as follows:
    30 $$\begin{aligned} \left. \left| \psi _3\right. \right\rangle&=D_p\ \left. \left| \psi _2\right. \right\rangle \nonumber \\&=a_q\sum _{r=0}^{2^n-1}{'' (}\left. \left| r\right. \right\rangle \otimes \ \left. \left| 0\right. \right\rangle )+b_q\sum _{r=0}^{2^n-1}{'\ (}\left. \left| r\right. \right\rangle \otimes \ \left. \left| 0\right. \right\rangle )\nonumber \\&\quad + c_q\sum _{r=0}^{2^n-1}{'\ (}\left. \left| r\right. \right\rangle \otimes \ \left. \left| 1\right. \right\rangle ). \end{aligned}$$
    The mean of the amplitudes to the illustrative example is
    31 $$\begin{aligned} \left\langle \alpha \right\rangle =\frac{\mathcal N-m}{{\mathcal {N}}\sqrt{{\mathcal {N}}}}=\frac{1}{8}, \end{aligned}$$
    then the inversions about the mean of the amplitudes^36^ are as follows:
    32 $$\begin{aligned} a_q=2*\left\langle \alpha \right\rangle -\frac{1}{\sqrt{{\mathcal {N}}}}\ =0,\ b_q{=}2*\left\langle \alpha \right\rangle =\frac{1}{4},\ c_q{=}-\frac{{1}}{\sqrt{{\mathcal {N}}}}=\frac{-1}{4}. \end{aligned}$$
    Hence, applying the partial diffusion $${D_p}$$ can take this form:
    33 $$\begin{aligned} \left. |\psi _3\right\rangle&={D_p}\left. |\psi _2\right\rangle ={D_p}\ I_{ f_1^T}\left( H^{\otimes 4}\otimes I\right) \left. |00000\right\rangle \nonumber \\&=\frac{1}{4}(\left. |0000\right\rangle +\left. |0001\right\rangle +\left. |0011\right\rangle +\left. |0101\right\rangle +\left. |0111\right\rangle \nonumber \\&\quad +\left. |1001\right\rangle +\left. |1011\right\rangle +\left. |1111\right\rangle )\otimes \left. |0\right\rangle \nonumber \\&\quad -\frac{1}{4}(\left. |0000\right\rangle +\left. |0001\right\rangle +\left. |0011\right\rangle +\left. |0101\right\rangle +\left. |0111\right\rangle \nonumber \\&\quad +\left. |1001\right\rangle +\left. |1011\right\rangle +\left. |1111\right\rangle )\otimes \left. |1\right\rangle .\end{aligned}$$
  - Apply the measurement on the auxiliary qubit, and if the outcome equals to one, we apply *Z* followed by *H* on the auxiliary qubit; otherwise, restart the previous steps. The probability to get $$\left. \left| 1\right. \right\rangle$$ on the auxiliary qubit is $${\mathcal {C}}|c_q|^2$$ and the superposition can be represented as follows:
    34 $$\begin{aligned} \left. \left| \psi _4\right. \right\rangle =\frac{1}{\sqrt{2^n}}\sum _ {f_{1}(r)=1}{(\left. \left| r\right. \right\rangle \otimes \left. \left| 1\right. \right\rangle ).} \end{aligned}$$
    Applying the measurement on the auxiliary qubit to the illustrative example is as follows:
    35 $$\begin{aligned} \left. |\psi _{4i}\right\rangle&=\frac{-\sqrt{2}}{4}(\left. |0000\right\rangle +\left. |0001\right\rangle +\left. |0011\right\rangle +\left. |0101\right\rangle \nonumber \\&\quad +\left. |0111\right\rangle +\left. |1001\right\rangle +\left. |1011\right\rangle +\left. |1111\right\rangle )\otimes \left. |1\right\rangle . \end{aligned}$$
    Applying *Z* on the auxiliary qubit to the illustrative example is as follows:
    36 $$\begin{aligned} \left. |\psi _{4ii}\right\rangle&=({I}^{\otimes 4}\otimes Z)\left. |\psi _{4i}\right\rangle \nonumber \\&=\frac{\sqrt{2}}{4}(\left. |0000\right\rangle +\left. |0001\right\rangle +\left. |0011\right\rangle +\left. |0101\right\rangle \nonumber \\&\quad +\left. |0111\right\rangle +\left. |1001\right\rangle +\left. |1011\right\rangle +\left. |1111\right\rangle )\otimes \left. |1\right\rangle . \end{aligned}$$
    Applying *H* on the auxiliary qubit to the illustrative example is as follows:
    37 $$\begin{aligned} \left. |\psi _{4iii}\right\rangle =&({I}^{\otimes 4}\otimes H)\left. |\psi _{4ii}\right\rangle =({I}^{\otimes 4}\otimes H)\otimes ({I}^{\otimes 4}\otimes Z)\left. |\psi _4i\right\rangle \nonumber \\ =&\frac{1}{4}(\left. |0000\right\rangle +\left. |0001\right\rangle +\left. |0011\right\rangle +\left. |0101\right\rangle +\left. |0111\right\rangle \nonumber \\&+\left. |1001\right\rangle +\left. |1011\right\rangle +\left. |1111\right\rangle )\otimes \left. |0\right\rangle \nonumber \\&-\frac{1}{4}(\left. |0000\right\rangle +\left. |0001\right\rangle +\left. |0011\right\rangle +\left. |0101\right\rangle +\left. |0111\right\rangle \nonumber \\&+\left. |1001\right\rangle +\left. |1011\right\rangle +\left. |1111\right\rangle )\otimes \left. |1\right\rangle . \end{aligned}$$
Apply the Arima algorithm^21^ as follows:
  Given: Phase oracles $$I_{ f_1^T}$$ and $$I_{ f_2^T}$$ and iterate the following for $$P = \left\lfloor {(\pi \sqrt{2{\mathcal {N}}}) }/{8}\right\rfloor$$ times to find one match and $$P = \left\lfloor {(\pi \sqrt{{\mathcal {N}}}) }/{8}\right\rfloor$$ times to find more than one match.
  - $$\left. |\psi {_5} \right\rangle = {\mathcal {R}}_{ f_2^T}\left. |\psi _4 \right\rangle$$
    When applying $${I_{ f_2^T}}$$ to the illustrative example, the form will be as follows:
    38 $$\begin{aligned} \left. |\psi _{5i}\right\rangle&=\ {I_{ f_2^T}} \left. |\psi _{4iii}\right\rangle \nonumber \\&=\frac{1}{4}(-\left. |0000\right\rangle +\left. |0001\right\rangle +\left. |0011\right\rangle +\left. |0101\right\rangle +\left. |0111\right\rangle \nonumber \\&\quad +\left. |1001\right\rangle +\left. |1011\right\rangle -\left. |1111\right\rangle )\otimes \left. |0\right\rangle \nonumber \\&\quad -\frac{1}{4}(-\left. |0000\right\rangle +\left. |0001\right\rangle +\left. |0011\right\rangle +\left. |0101\right\rangle +\left. |0111\right\rangle \nonumber \\&\quad +\left. |1001\right\rangle +\left. |1011\right\rangle -\left. |1111\right\rangle )\otimes \left. |1\right\rangle . \end{aligned}$$
    The mean of the amplitudes to the illustrative example is
    39 $$\begin{aligned} \left\langle \alpha \right\rangle =\frac{6*\frac{1}{4}-2*\frac{1}{4}+8*0}{16}=\frac{1}{16}, \end{aligned}$$
    then, the inversions about the mean of the amplitudes are as follows^21^:
    40 $$\begin{aligned} -\beta +2*\left\langle \alpha \right\rangle&=\frac{-1}{4}+2*\frac{1}{16}=\frac{-1}{8}, \end{aligned}$$
    41 $$\begin{aligned} -\alpha +2*\left\langle \alpha \right\rangle&=\frac{1}{4}+2*\frac{1}{16}=\frac{3}{8}, \end{aligned}$$
    42 $$\begin{aligned} -\gamma +2*\left\langle \alpha \right\rangle&=0+2*\frac{1}{16}=\frac{1}{8}, \end{aligned}$$
    such that $$\beta$$ represents the amplitude of any state in the stored data, but it is not the desired element, $$\alpha$$ represents the amplitude of the state of the desired element, and $$\gamma$$ represents the amplitude of any state not in the stored data and not be the desired element.
    Hence, applying the Grover operator *G* can take this form:
    43 $$\begin{aligned} \left. |\psi _{5ii}\right\rangle&=G\left. |\psi _{5i}\right\rangle \nonumber \\&=\frac{-1}{8}({-3}\left. |0000\right\rangle +\left. |0001\right\rangle -\left. |0010\right\rangle +\left. |0011\right\rangle -\left. |0100\right\rangle \nonumber \\&\quad +\left. |0101\right\rangle -\left. |0110\right\rangle +\left. |0111\right\rangle -\left. |1000\right\rangle +\left. |1001\right\rangle -\left. |1010\right\rangle \nonumber \\&\quad +\left. |1011\right\rangle -\left. |1100\right\rangle -\left. |1101\right\rangle -\left. |1110\right\rangle -{3}\left. |1111\right\rangle )\otimes \left. |0\right\rangle \nonumber \\&\quad +\frac{1}{8}( {-3}\left. |0000\right\rangle +\left. |0001\right\rangle -\left. |0010\right\rangle +\left. |0011\right\rangle -\left. |0100\right\rangle \nonumber \\&\quad +\left. |0101\right\rangle -\left. |0110\right\rangle +\left. |0111\right\rangle -\left. |1000\right\rangle +\left. |1001\right\rangle -\left. |1010\right\rangle \nonumber \\&\quad +\left. |1011\right\rangle -\left. |1100\right\rangle -\left. |1101\right\rangle -\left. |1110\right\rangle -{3}\left. |1111\right\rangle )\otimes \left. |1\right\rangle . \end{aligned}$$
  - $$\left. |\psi {_6} \right\rangle ={\mathcal {R}}_{ f_1^T}\left. |\psi {_5}\right\rangle$$.
    When applying $${I_{ f_1^T}}$$ to the illustrative example, the form will be as follows:
    44 $$\begin{aligned} \left. |\psi _{6i}\right\rangle&={I_{ f_1^T}}\left. |\psi _{5ii}\right\rangle \nonumber \\&=\frac{1}{8}({-3}\left. |0000\right\rangle +\left. |0001\right\rangle +\left. |0010\right\rangle +\left. |0011\right\rangle +\left. |0100\right\rangle \nonumber \\&\quad +\left. |0101\right\rangle +\left. |0110\right\rangle +\left. |0111\right\rangle +\left. |1000\right\rangle +\left. |1001\right\rangle +\left. |1010\right\rangle \nonumber \\&\quad +\left. |1011\right\rangle +\left. |1100\right\rangle +\left. |1101\right\rangle +\left. |1110\right\rangle -{3}\left. |1111\right\rangle )\otimes \left. |0\right\rangle \nonumber \\&\quad +\frac{-1}{8}( {-3}\left. |0000\right\rangle +\left. |0001\right\rangle +\left. |0010\right\rangle +\left. |0011\right\rangle +\left. |0100\right\rangle \nonumber \\&\quad +\left. |0101\right\rangle +\left. |0110\right\rangle +\left. |0111\right\rangle +\left. |1000\right\rangle +\left. |1001\right\rangle +\left. |1010\right\rangle \nonumber \\&\quad +\left. |1011\right\rangle +\left. |1100\right\rangle +\left. |1101\right\rangle +\left. |1110\right\rangle -{3}\left. |1111\right\rangle )\otimes \left. |1\right\rangle . \end{aligned}$$
    $$\left\langle \alpha \right\rangle = \frac{{2*\frac{{ - 3}}{8} + 6*\frac{1}{8} + 8*\frac{1}{8}}}{{16}} = \frac{1}{{16}}$$for the amplitudes of states to the illustrative example which product to the state $$\left. |0\right\rangle$$. Then, the inversions of the mean for the amplitudes of this states are as follows:
    45 $$\begin{aligned} -\alpha +2*\left\langle \alpha \right\rangle&=\frac{3}{8}+2*\frac{1}{16}=\frac{1}{2}, \end{aligned}$$
    46 $$\begin{aligned} -\beta +2*\left\langle \alpha \right\rangle&=\frac{-1}{8}+2*\frac{1}{16}=0, \end{aligned}$$
    47 $$\begin{aligned} -\gamma +2*\left\langle \alpha \right\rangle&=\frac{-1}{8}+2*\frac{1}{16}=0, \end{aligned}$$
    and $$\left\langle \alpha \right\rangle =\frac{2*\frac{3}{8}+6*\frac{-1}{8}+8*\frac{-1}{8}}{16}=\frac{-1}{16}$$ for the amplitudes of states to the illustrative example which product to the state $$\left. |1\right\rangle$$, then, the inversions of the mean for amplitudes of this states are as follows:
    48 $$\begin{aligned} -\alpha +2*\left\langle \alpha \right\rangle&=\frac{-3}{8}+2*\frac{-1}{16}=\frac{-1}{2}, \end{aligned}$$
    49 $$\begin{aligned} -\beta +2*\left\langle \alpha \right\rangle&=\frac{1}{8}+2*\frac{-1}{16}=0, \end{aligned}$$
    50 $$\begin{aligned} -\gamma +2*\left\langle \alpha \right\rangle&=\frac{1}{8}+2*\frac{-1}{16}=0 \end{aligned}$$
    Hence, applying the Grover operator *G* can take this form:
    51 $$\begin{aligned} \left. |\psi _{6ii}\right\rangle =&G \left. |\psi _{6i}\right\rangle \nonumber \\ =&\frac{1}{2}\left( \left. |0000\right\rangle +\left. |1111\right\rangle \right) \otimes \left. |0\right\rangle -\frac{1}{2}\left( \left. |0000\right\rangle +\left. |1111\right\rangle \right) \otimes \left. |1\right\rangle .\ \end{aligned}$$
  - Observe the system..
3. Suppose that the system $$\left. \left| {{\psi }}_{{7}}\right. \right\rangle =\alpha _{i}(\left. \left| { r}\right. \right\rangle \otimes \left. \left| 1\right. \right\rangle )+\beta _{i}(\left. \left| { r}\right. \right\rangle \otimes \left. \left| { 0}\right. \right\rangle )$$ and to find the probability of a match out of the *R* possible match between $${\mathcal {N}}$$ items as follows
  52 $$\begin{aligned} q={R}*|\alpha _{i}|^2. \end{aligned}$$
  To the illustrative example, the intersection that makes $$f_{{1}}$$ and $$f_{{ 2}}$$ evaluate to True can be obtained with the probability $$2*{\left( \frac{-1}{2}\right) }^2=0.50$$ for $$\left. |0\right\rangle$$ or $$\left. |15\right\rangle$$.

Arima algorithm can solve the illustrative example by searching in $f_1$, supposing that the searching data are $\left. |0\right\rangle$ and $\left. |15\right\rangle$ and $P=1$. In order to save space, instead of writing out the entire superposition of states, a transposed vector of coefficients will be used, where the 16 basis states index the vector.

The initial state described by $|\psi _{0}\rangle =\frac{1}{2\sqrt{2}}(1, 1, 0, 1, 0, 1, 0, 1, 0, 1, 0, 1, 0, 0, 0, 1).$

$$|\psi {_1}\rangle ={I_{ f_1^T}}|\psi _{0}\rangle =\frac{1}{2 \sqrt{2}}(-1, 1, 0, 1, 0, 1, 0, 1, 0, 1, 0, 1, 0, 0, 0, -1).$$

$$|\psi {_2}\rangle = G|\psi {_1}\rangle =\frac{1}{4 \sqrt{2}}(3, -1, 1, -1, 1, -1, 1, -1, 1, -1, 1, -1, 1, 1, 1, 3).$$

$$|\psi {_3}\rangle ={I_{ f_2^T}}|\psi _{2}\rangle =\frac{1}{4 \sqrt{2}}(-3, 1, 1, 1, 1, 1, 1, 1, 1, 1, 1, 1, 1, 1, 1, -3).$$

$$|\psi _{4}\rangle =G|\psi {_3}\rangle =\frac{1}{\sqrt{2}}(1, 0, 0, 0, 0, 0, 0, 0, 0, 0, 0, 0, 0, 0, 0, 1).$$

**Figure 1:**
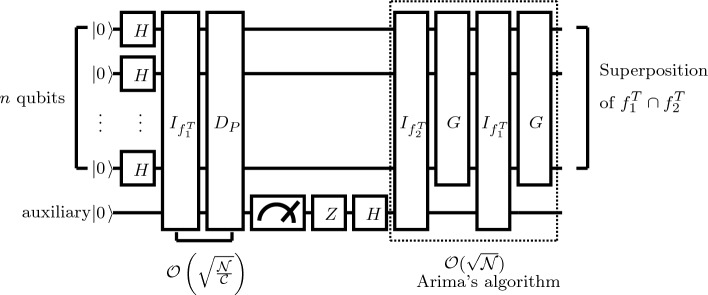
Quantum circuit for the proposed true intersection algorithm.

Then the probability $={\left( \frac{{ 1}}{\sqrt{{ 2}}}\right) }^{{ 2}}{ =}$ 0.50 for either state $\left. |0\right\rangle$ or $\left. |15\right\rangle$ as the same result as the proposed algorithm. A pseudocode of true intersection operation is shown in Algorithm 1; the circuit of the proposed algorithm is shown in Fig. 1.

**Algorithm 1:**
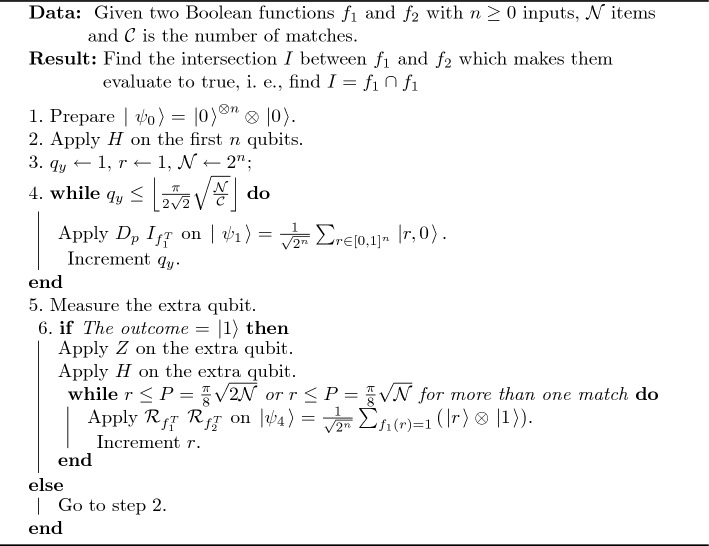
An Algorithm of True Intersection Operation.

### The proposed quantum algorithm for false intersection operation

Given two Boolean functions $f_1$ and ${f}_2$ it is required to find the set of binary vectors that evaluates both $f_1\oplus 1$ ^42^ and ${f}_2\oplus 1$ to True at the same time, i. e., to find the false intersection between them, the steps of the proposed algorithm will be illustrated using the illustrative example:

1. Apply Younes et al. algorithm^36^ as follows:
  - The preparation of the register consists of $$n+1$$ qubits, and all of them are in the state $$\left. |0\right\rangle$$. The auxiliary qubit is used to evaluate the Boolean function $$f_1$$. The state of the system is as in Eqs. (22) and (23).
  - The initialization of the register in which the Hadamard gate *H* is applied on the first *n* qubits in parallel as in Eqs. (24) and (25).
  - This algorithm iterates the following steps for $$\left\lfloor \frac{\pi }{2\sqrt{2}}\sqrt{\frac{{\mathcal {N}}}{\mathcal C}}\right\rfloor$$
    (i) Apply the oracle operator $$I_{ f_1^T}$$ on $$n+1$$ qubits where $$I_{ f_1^T}$$ evaluates the first boolean function $$f_1$$ as in Eqs. (26), (27) , and (28).
    (ii) Apply *X* on the auxiliary qubit^42^as follows in Eq. (53).
    53 $$\begin{aligned} \left. \left| \psi _3\right. \right\rangle&=(I_n\otimes X)\otimes \left. \left| \psi _2\right. \right\rangle .\nonumber \\&=\frac{1}{\sqrt{2^n}}\sum ^{{{2}}^n-{ 1}}_{r{ =0}}(I_n\otimes X)\otimes \left. \left| r,f_1(r)\right. \right\rangle . \end{aligned}$$
    When applying Eq. (53) to the illustrative example, the form will be as follows:
    54 $$\begin{aligned} \left. |\psi _3\right\rangle&=\left( I^{\otimes 4}\otimes X\right) \left. |\psi _2\right\rangle \nonumber \\&=\frac{1}{4}(\left. |00000\right\rangle +\left. |00010\right\rangle +\left. |00101\right\rangle +\left. |00110\right\rangle +\left. |01001\right\rangle \nonumber \\&\quad +\left. |01010\right\rangle +\left. |01101\right\rangle +\left. |01110\right\rangle +\left. |10001\right\rangle +\left. |10010\right\rangle \nonumber \\&\quad + \left. |10101\right\rangle +\left. |10110\right\rangle +\left. |11001\right\rangle +\left. |11011\right\rangle +\left. |11101\right\rangle \nonumber \\&\quad +\left. |11110\right\rangle ). \end{aligned}$$
    (iii) After that, the algorithm applies the partial diffusion $$D_p$$ on the $$n+1$$ qubits as follows:
    55 $$\begin{aligned} \left. \left| \psi _4\right. \right\rangle&=D_p\ \left. \left| \psi _3\right. \right\rangle \nonumber \\&=a_q\sum _{r=0}^{2^n-1}{' (}\left. \left| r\right. \right\rangle \otimes \ \left. \left| 0\right. \right\rangle )+b_q\sum _{r=0}^{2^n-1}{''\ (}\left. \left| r\right. \right\rangle \otimes \ \left. \left| 0\right. \right\rangle )\nonumber \\&\quad + c_q\sum _{r=0}^{2^n-1}{''\ (}\left. \left| r\right. \right\rangle \otimes \ \left. \left| 1\right. \right\rangle ). \end{aligned}$$
    The mean of amplitudes to the illustrative example is
    56 $$\begin{aligned} \left\langle \alpha \right\rangle =\frac{{\mathcal {N}}-m}{N\sqrt{{\mathcal {N}}}}=\frac{1}{8}, \end{aligned}$$
    then the inversions about the mean of the amplitudes are
    57 $$\begin{aligned} a_q=2*\left\langle \alpha \right\rangle -\frac{1}{\sqrt{{\mathcal {N}}}}\ =0,\ b_q{=}2*\left\langle \alpha \right\rangle =\frac{1}{4},\ c_q{=}-\frac{{ 1}}{\sqrt{{\mathcal {N}}}}=\frac{-1}{4}. \end{aligned}$$
    Hence, applying the partial diffusion $$D_p$$ can take this form
    58 $$\begin{aligned} \left. |\psi _4\right\rangle&={D_p}\left. |\psi _3\right\rangle =\frac{1}{4}(\left. |0010\right\rangle +\left. |0100\right\rangle +\left. |0110\right\rangle +\left. |1000\right\rangle +\left. |1010\right\rangle \nonumber \\&\quad +\left. |1100\right\rangle +\left. |1101\right\rangle +\left. |1110\right\rangle )\otimes \left. |0\right\rangle \nonumber \\&\quad -\frac{1}{4}(\left. |0010\right\rangle +\left. |0100\right\rangle +\left. |0110\right\rangle +\left. |1000\right\rangle +\left. |1010\right\rangle \nonumber \\&\quad +\left. |1100\right\rangle +\left. |1101\right\rangle +\left. |1110\right\rangle )\otimes \left. |1\right\rangle . \end{aligned}$$
  - Apply the measurement on the auxiliary qubit, and if the outcome equals to one, we apply *Z* followed by *H* on the auxiliary qubit; otherwise, restart the previous steps. The probability to get $$\left. \left| 1\right. \right\rangle$$ on the auxiliary qubit is $${\mathcal {C}}|c_q|^2$$ and the superposition can be represented as follows:
    59 $$\begin{aligned} \left. \left| \psi _5\right. \right\rangle =\frac{1}{\sqrt{2^n}}\sum _ {f_{1}(r)=0}{(\left. \left| r\right. \right\rangle \otimes \left. \left| 1\right. \right\rangle ).} \end{aligned}$$
    Applying the measurement on the auxiliary qubit to the illustrative example is as follows:
    60 $$\begin{aligned} \left. |\psi _{5i}\right\rangle&=\frac{-\sqrt{2}}{4}(\left. |0010\right\rangle +\left. |0100\right\rangle +\left. |0110\right\rangle +\left. |1000\right\rangle +\left. |1010\right\rangle \nonumber \\&\quad +\left. |1100\right\rangle +\left. |1101\right\rangle +\left. |1110\right\rangle )\otimes \left. |1\right\rangle . \end{aligned}$$
    Applying *Z* on the auxiliary qubit to the illustrative example is as follows:
    61 $$\begin{aligned} \left. |\psi _{5ii}\right\rangle&=\left( I^{\otimes 4}\otimes Z\right) \left. |\psi _{5i}\right\rangle \nonumber \\&=\frac{\sqrt{2}}{4}(\left. |0010\right\rangle +\left. |0100\right\rangle +\left. |0110\right\rangle +\left. |1000\right\rangle +\left. |1010\right\rangle \nonumber \\&\quad +\left. |1100\right\rangle +\left. |1101\right\rangle +\left. |1110\right\rangle )\otimes \left. |1\right\rangle . \end{aligned}$$
    Applying *H* on the auxiliary qubit to the illustrative example is as follows:
    62 $$\begin{aligned} \left. |\psi _{5iii}\right\rangle&=\left( I^{\otimes 4}\otimes H\right) \left. |\psi _{5ii}\right\rangle \nonumber \\&=\frac{1}{4}(\left. |0010\right\rangle +\left. |0100\right\rangle +\left. |0110\right\rangle +\left. |1000\right\rangle +\left. |1010\right\rangle \nonumber \\&\quad +\left. |1100\right\rangle +\left. |1101\right\rangle 
+\left. |1110\right\rangle )\otimes \left. |0\right\rangle \nonumber \\&\quad -\frac{1}{4}(\left. |0010\right\rangle +\left. |0100\right\rangle +\left. |0110\right\rangle +\left. |1000\right\rangle +\left. |1010\right\rangle \nonumber \\&\quad +\left. |1100\right\rangle +\left. |1101\right\rangle +\left. |1110\right\rangle )\otimes \left. |1\right\rangle . \end{aligned}$$
2. Apply the Arima algorithm as follows
  Given: Phase oracles $$I_{ f_1^T}$$* and *$$I_{ f_2^T}$$ and iterate for $$P = \left\lfloor {(\pi \sqrt{2{\mathcal {N}}}) }/{8}\right\rfloor$$ times to find one match and $$P = \left\lfloor {(\pi \sqrt{{\mathcal {N}}}) }/{8}\right\rfloor$$ times to find more than one match the following
  - $$\left. \left| \psi _6\right. \right\rangle = I_{ f_2^T}\left. \left| \psi _5\right. \right\rangle$$.
    When applying $$I_{ f_2^T}$$ to the illustrative example, the form will be as follows:
    63 $$\begin{aligned} \left. |\psi _6\right\rangle&=I_{ f_2^T}\left. |\psi _{5iii}\right\rangle \nonumber \\ {}&= -\frac{1}{4}(\left. |0010\right\rangle +\left. |0100\right\rangle +\left. |0110\right\rangle +\left. |1000\right\rangle +\left. |1010\right\rangle \nonumber \\&\quad +\left. |1100\right\rangle -\left. |1101\right\rangle -\left. |1110\right\rangle )\otimes \left. |0\right\rangle \nonumber \\&\quad +\frac{1}{4}(\left. |0010\right\rangle +\left. |0100\right\rangle +\left. |0110\right\rangle +\left. |1000\right\rangle +\left. |1010\right\rangle \nonumber \\&\quad +\left. |1100\right\rangle -\left. |1101\right\rangle -\left. |1110\right\rangle )\otimes \left. |1\right\rangle . \end{aligned}$$
  - $$\left. \left| \psi _7\right. \right\rangle =({I_n\otimes X})\left. \left| \psi _6\right. \right\rangle$$.
    Applying *X* on the auxiliary qubit to the illustrative example is as follows:
    64 $$\begin{aligned} \left. |\psi _7\right\rangle&=\left( I^{\otimes 4}\otimes X\right) \left. |\psi _6\right\rangle \nonumber \\&=\frac{1}{4}(\left. |0010\right\rangle +\left. |0100\right\rangle +\left. |0110\right\rangle +\left. |1000\right\rangle +\left. |1010\right\rangle \nonumber \\&\quad +\left. |1100\right\rangle -\left. |1101\right\rangle -\left. |1110\right\rangle )\otimes \left. |0\right\rangle \nonumber \\&\quad -\frac{1}{4}(\left. |0010\right\rangle +\left. |0100\right\rangle +\left. |0110\right\rangle +\left. |1000\right\rangle +\left. |1010\right\rangle \nonumber \\&\quad +\left. |1100\right\rangle -\left. |1101\right\rangle -\left. |1110\right\rangle )\otimes \left. |1\right\rangle . \end{aligned}$$
  - $$\left. \left| \psi _8\right. \right\rangle ={ G}\left. \left| \psi _7\right. \right\rangle$$.
    The mean of the amplitudes to the illustrative example is
    65 $$\begin{aligned} \left\langle \alpha \right\rangle =\frac{6*\frac{1}{4}-2*\frac{1}{4}+8*0}{16}=\frac{1}{16}, \end{aligned}$$
    then, the inversions about the mean of the amplitudes for states are as follows:
    66 $$\begin{aligned} -\beta +2*\left\langle \alpha \right\rangle&=\frac{-1}{4}+2*\frac{1}{16}=\frac{-1}{8}, \end{aligned}$$
    67 $$\begin{aligned} -\alpha +2*\left\langle \alpha \right\rangle&=\frac{1}{4}+2*\frac{1}{16}=\frac{3}{8}, \end{aligned}$$
    68 $$\begin{aligned} -\gamma +2*\left\langle \alpha \right\rangle&=0+2*\frac{1}{16}=\frac{1}{8}. \end{aligned}$$
    Hence, applying the Grover operator *G* can take this form:
    69 $$\begin{aligned} \left. |\psi _8\right\rangle&= G \left. |\psi _{7}\right\rangle \nonumber \\&= -\frac{1}{8}(-\left. |0000\right\rangle -\left. |0001\right\rangle +\left. |0010\right\rangle -\left. |0011\right\rangle +\left. |0100\right\rangle \nonumber \\&\quad -\left. |0101\right\rangle +\left. |0110\right\rangle -\left. |0111\right\rangle +\left. |1000\right\rangle -\left. |1001\right\rangle \nonumber \\&\quad +\left. |1010\right\rangle -\left. |1011\right\rangle +\left. |1100\right\rangle - 3\left. |1101\right\rangle -3\left. |1110\right\rangle \nonumber \\&\quad -\left. |1111\right\rangle )\otimes \left. |0\right\rangle \nonumber \\&\quad +\frac{1}{8}(-\left. |0000\right\rangle -\left. |0001\right\rangle +\left. |0010\right\rangle -\left. |0011\right\rangle +\left. |0100\right\rangle \nonumber \\&\quad -\left. |0101\right\rangle +\left. |0110\right\rangle -\left. |0111\right\rangle +\left. |1000\right\rangle -\left. |1001\right\rangle \nonumber \\&\quad +\left. |1010\right\rangle -\left. |1011\right\rangle +\left. |1100\right\rangle - 3\left. |1101\right\rangle -3\left. |1110\right\rangle \nonumber \\&\quad -\left. |1111\right\rangle )\otimes \left. |1\right\rangle . \end{aligned}$$
  - $$\left. |\psi {_9} \right\rangle ={\mathcal {R}}_{ f_1^T}\left. |\psi {_8}\right\rangle$$.
    When applying $${I_{ f_1^T}}$$ to the illustrative example, the form will be as follows:
    70 $$\begin{aligned} \left. |\psi _{9i}\right\rangle&= {I_{ f_1^T}}\left. |\psi _{8}\right\rangle \nonumber \\&= -\frac{1}{8}(\left. |0000\right\rangle +\left. |0001\right\rangle +\left. |0010\right\rangle +\left. |0011\right\rangle +\left. |0100\right\rangle \nonumber \\&\quad +\left. |0101\right\rangle +\left. |0110\right\rangle +\left. |0111\right\rangle +\left. |1000\right\rangle +\left. |1001\right\rangle \nonumber \\&\quad +\left. |1010\right\rangle +\left. |1011\right\rangle +\left. |1100\right\rangle - 3\left. |1101\right\rangle -3\left. |1110\right\rangle \nonumber \\&\quad +\left. |1111\right\rangle )\otimes \left. |0\right\rangle \nonumber \\&\quad +\frac{1}{8}(\left. |0000\right\rangle +\left. |0001\right\rangle +\left. |0010\right\rangle +\left. |0011\right\rangle +\left. |0100\right\rangle \nonumber \\&\quad +\left. |0101\right\rangle +\left. |0110\right\rangle +\left. |0111\right\rangle +\left. |1000\right\rangle +\left. |1001\right\rangle \nonumber \\&\quad +\left. |1010\right\rangle +\left. |1011\right\rangle +\left. |1100\right\rangle - 3\left. |1101\right\rangle -3\left. |1110\right\rangle \nonumber \\&\quad +\left. |1111\right\rangle )\otimes \left. |1\right\rangle \end{aligned}$$
    $$\left\langle \alpha \right\rangle = \frac{{2*\frac{3}{8} + 6*\frac{{ - 1}}{8} + 8*\frac{{ - 1}}{8}}}{{16}} = \frac{{ - 1}}{{16}}$$ for the amplitudes of states to the illustrative example which product to the state $$\left. |0\right\rangle$$. Then, the inversions of the mean for the amplitudes of these states are as follows:
    71 $$\begin{aligned} -\alpha +2*\left\langle \alpha \right\rangle&=\frac{-3}{8}+2*\frac{-1}{16}=\frac{-1}{2}, \end{aligned}$$
    72 $$\begin{aligned} -\beta +2*\left\langle \alpha \right\rangle&=\frac{1}{8}+2*\frac{-1}{16}=0, \end{aligned}$$
    73 $$\begin{aligned} -\gamma +2*\left\langle \alpha \right\rangle&=\frac{1}{8}+2*\frac{-1}{16}=0, \end{aligned}$$
    and $$\left\langle \alpha \right\rangle = \frac{{2*\frac{{ - 3}}{8} + 6*\frac{1}{8} + 8*\frac{1}{8}}}{{16}} = \frac{1}{{16}}$$ for the amplitudes of states to the illustrative example which product to the state $$\left. |1\right\rangle$$. Then, the inversions of the mean for amplitudes of these states are as follows:
    74 $$\begin{aligned} -\alpha +2*\left\langle \alpha \right\rangle&=\frac{3}{8}+2*\frac{1}{16}=\frac{1}{2}, \end{aligned}$$
    75 $$\begin{aligned} -\beta +2*\left\langle \alpha \right\rangle&=\frac{-1}{8}+2*\frac{1}{16}=0, \end{aligned}$$
    76 $$\begin{aligned} -\gamma +2*\left\langle \alpha \right\rangle&=\frac{-1}{8}+2*\frac{1}{16}=0, \end{aligned}$$
    Hence, applying the Grover operator *G* can take this form
    77 $$\begin{aligned} \left. |\psi _{9ii}\right\rangle&\nonumber = G\left. |\psi _{9i}\right\rangle \\&=\frac{-1}{2}\left( \left. |1101\right\rangle +\left. |1110\right\rangle \right) \otimes \left. |0\right\rangle +\frac{1}{2}\left( \left. |1101\right\rangle +\left. |1110\right\rangle \right) \otimes \left. |1\right\rangle . \end{aligned}$$
  - Observe the system.
3. Find the probability of a match out of the *R* possible match between $${\mathcal {N}}$$ items as in Eq. (52).

To the illustrative example, the intersection that makes $f_{{1}}$ and $f_{{ 2}}$ evaluate to False can be obtained with the probability $2*{\left( \frac{1}{2}\right) }^2=0.50$ for $\left. |13\right\rangle$ or $\left. |14\right\rangle$.

Arima algorithm with an adjustment by applying *X* after applying *H* can solve the illustrative example by searching for the false assignment of $f_1$. Suppose that searching data $\left. |13\right\rangle$ and $\left. |14\right\rangle$ where $P=1$. The initial state described by $|\psi _{0}\rangle =\frac{1}{2\sqrt{2}}(1, 1, 0, 1, 0, 1, 0, 1, 0, 1, 0,1 ,0 ,0 ,0 ,1).$

$$|\psi _{1}\rangle =\left( I^{\otimes 4}\otimes X\right) |\psi _{0}\rangle =\frac{1}{2\sqrt{2}} (0, 0, 1, 0, 1, 0, 1, 0, 1, 0, 1, 0, 1, 1, 1, 0).$$

$$|\psi _{2}\rangle ={I_{ f_1^T}}|\psi _{1}\rangle =\frac{1}{2\sqrt{2}} (0, 0, 1, 0, 1, 0, 1, 0, 1, 0, 1, 0, 1, -1, -1, 0).$$

$$|\psi _{3}\rangle = G|\psi _{2}\rangle =\frac{1}{4\sqrt{2}} (1, 1, -1, 1, -1, 1, -1, 1, -1, 1, -1, 1, - 1, 3, 3, 1).$$

$$|\psi _{4}\rangle ={I_{ f_2^T}}|\psi _{3}\rangle =\frac{1}{4\sqrt{2}} (-1, -1, -1, -1, -1, -1, -1, -1, -1, -1, -1, -1, -1, 3, 3, -1)$$.

$$|\psi _{5}\rangle = G|\psi _{4}\rangle =\frac{1}{\sqrt{2}}\ (0, 0, 0, 0, 0, 0, 0, 0, 0, 0, 0, 0, 0, -1, -1, 0).$$

Then the probability $= \left( {\frac{{ - 1}}{{\sqrt 2 }}} \right)^{2} = 0.50$ for any either $\left. |13\right\rangle$ or$\left. |14\right\rangle$ as the same result of the proposed algorithm.

**Figure 2:**
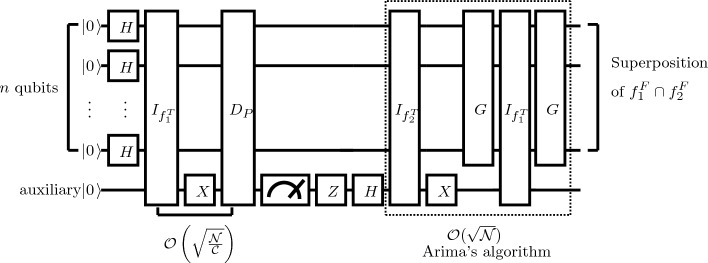
Quantum circuit for the proposed false intersection algorithm.

A pseudocode of false intersection operation is shown in Algorithm 2; the circuit of the proposed algorithm is shown in Fig. 2.

**Algorithm 2:**
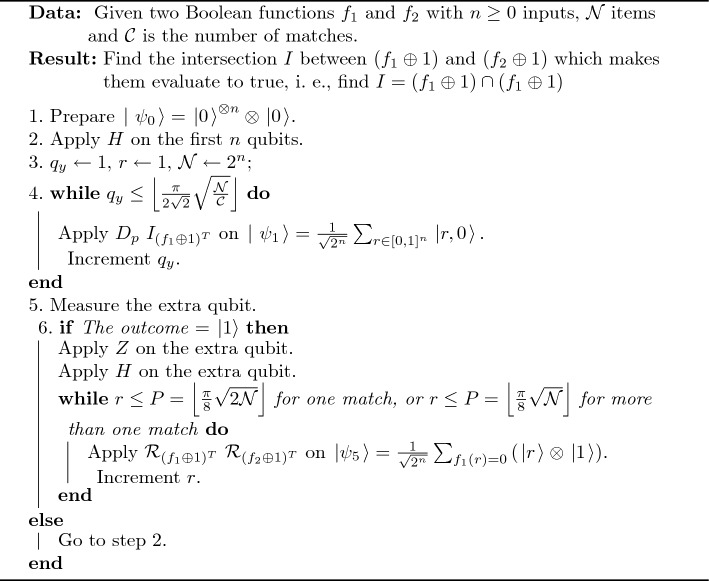
An Algorithm of False Intersection Operation.

### The proposed quantum algorithm for difference operation

Given two Boolean functions ${f_{{1}}}$ and ${f_{{ 2}}}$, it is required to find the set of binary vectors that evaluates ${f_{{1}}}$ to True and evaluates ${f_{{ 2}}}$ to False, i. e., to find the difference between ${f_{{1}}}$ and ${f_{{ 2}}}$, and the steps of the proposed algorithm will be illustrated using the illustrative example:

1. Apply Younes et al. algorithm as follows:
  - The preparation of the register consists of $n+1$ qubits, and all of them are in the state $\left. |0\right\rangle$. The auxiliary qubit is used to evaluate the Boolean function $f_1$. The state of the system is as in Eq. (22) and Eq. (23).
  - The initialization of the register in which the Hadamard gate *H* is applied on the first *n* qubits in parallel as in Eqs. (24) and (25).
  - This algorithm iterates the following steps for $\left\lfloor \frac{\pi }{2\sqrt{2}}\sqrt{\frac{{\mathcal {N}}}{\mathcal C}}\right\rfloor$ Apply the oracle operator $I_{ f_1^T}$ on $n+1$ qubits where $I_{ f_1^T}$ evaluates the first boolean function $f_1$ as in equations (26), (27), and (28). After that, the algorithm applies the partial diffusion $D_p$ on the $n+1$ qubits as follows in Eqs. (29) and (33):
  - Apply the measurement on the auxiliary qubit, and if the outcome equals to one, we apply *Z* followed by *H* on the auxiliary qubit; otherwise, restart the previous steps. The probability of getting $\left. \left| 1\right. \right\rangle$ on the auxiliary qubit is ${\mathcal {C}}|c_q|^2$, and the superposition can be represented as in Eqs. (34-37).
2. Apply the Arima algorithm for $P = \left\lfloor {(\pi \sqrt{2\mathcal N}) }/{8}\right\rfloor$ times to find difference as follows:
  - $$\left. \left| \psi _5\right. \right\rangle = I_{ f_2^T}\left. \left| \psi _4\right. \right\rangle$$.
    When applying $I_{ f_2^T}$ to the illustrative example, the form will be as follows:
    78 $$\begin{aligned} \left. |\psi _5\right\rangle&={I_{ f_2^T}}\left. |\psi _{4iii}\right\rangle \nonumber \\&=\frac{1}{4}(-\left. |0000\right\rangle +\left. |0001\right\rangle +\left. |0011\right\rangle +\left. |0101\right\rangle +\left. |0111\right\rangle \nonumber \\&\quad +\left. |1001\right\rangle +\left. |1011\right\rangle -\left. |1111\right\rangle )\otimes \left. |0\right\rangle \nonumber \\&\quad -\frac{1}{4}(-\left. |0000\right\rangle +\left. |0001\right\rangle +\left. |0011\right\rangle +\left. |0101\right\rangle +\left. |0111\right\rangle \nonumber \\&\quad +\left. |1001\right\rangle +\left. |1011\right\rangle -\left. |1111\right\rangle )\otimes \left. |1\right\rangle . \end{aligned}$$
  - $$\left. \left| \psi _6\right. \right\rangle =({I_n\otimes X})\left. \left| \psi _5\right. \right\rangle$$.
    Applying *X* on the auxiliary qubit to the illustrative example is as follows
    79 $$\begin{aligned} \left. |\psi _6\right\rangle&=\left( I^{\otimes 4}\otimes X\right) \left. |\psi _5\right\rangle \nonumber \\&=\frac{-1}{4}(-\left. |0000\right\rangle +\left. |0001\right\rangle +\left. |0011\right\rangle +\left. |0101\right\rangle +\left. |0111\right\rangle \nonumber \\&\quad +\left. |1001\right\rangle +\left. |1011\right\rangle -\left. |1111\right\rangle )\otimes \left. |0\right\rangle \nonumber \\&\quad +\frac{1}{4}(-\left. |0000\right\rangle +\left. |0001\right\rangle +\left. |0011\right\rangle +\left. |0101\right\rangle +\left. |0111\right\rangle \nonumber \\&\quad +\left. |1001\right\rangle +\left. |1011\right\rangle -\left. |1111\right\rangle )\otimes \left. |1\right\rangle . \end{aligned}$$
  - $$\left. \left| \psi _7\right. \right\rangle ={ G}\left. \left| \psi _6\right. \right\rangle$$.
    The mean of the amplitudes to the illustrative example is
    80 $$\begin{aligned} \left\langle \alpha \right\rangle =\frac{6*\frac{-1}{4}+2*\frac{1}{4}+8*0}{16}=\frac{-1}{16}, \end{aligned}$$
    then, the inversions about the mean of the amplitudes of states are as follows:
    81 $$\begin{aligned} -\beta +2*\left\langle \alpha \right\rangle&=\frac{-1}{4}+2*\frac{-1}{16}=\frac{-3}{8}, \end{aligned}$$
    82 $$\begin{aligned} -\alpha +2*\left\langle \alpha \right\rangle&=\frac{1}{4}+2*\frac{-1}{16}=\frac{1}{8}, \end{aligned}$$
    83 $$\begin{aligned} -\gamma +2*\left\langle \alpha \right\rangle&=0+2*\frac{-1}{16}=\frac{-1}{8}, \end{aligned}$$
    Hence, applying the Grover operator *G* can take this form:
    84 $$\begin{aligned} \left. |\psi _7\right\rangle&=G\left. |\psi _6\right\rangle \nonumber \\&=\frac{1}{8}(-3\left. |0000\right\rangle +\left. |0001\right\rangle -\left. |0010\right\rangle +\left. |0011\right\rangle -\left. |0100\right\rangle \nonumber \\&\quad +\left. |0101\right\rangle -\left. |0110\right\rangle +\left. |0111\right\rangle -\left. |1000\right\rangle +\left. |1001\right\rangle \nonumber \\&\quad -\left. |1010\right\rangle +\left. |1011\right\rangle -\left. |1100\right\rangle -\left. |1101\right\rangle -\left. |1110\right\rangle \nonumber \\&\quad -3\left. |1111\right\rangle )\otimes \left. |0\right\rangle \nonumber \\&\quad -\frac{1}{8}(-3\left. |0000\right\rangle +\left. |0001\right\rangle -\left. |0010\right\rangle +\left. |0011\right\rangle -\left. |0100\right\rangle \nonumber \\&\quad +\left. |0101\right\rangle -\left. |0110\right\rangle +\left. |0111\right\rangle -\left. |1000\right\rangle +\left. |1001\right\rangle \nonumber \\&\quad -\left. |1010\right\rangle +\left. |1011\right\rangle -\left. |1100\right\rangle -\left. |1101\right\rangle -\left. |1110\right\rangle \nonumber \\&\quad -3\left. |1111\right\rangle )\otimes \left. |1\right\rangle . \end{aligned}$$
  - $$\left. |\psi {_8} \right\rangle ={\mathcal {R}}_{ f_1^T}\left. |\psi {_7}\right\rangle$$.
    When applying ${I_{ f_1^T}}$ to the illustrative example, the form will be as follows:
    85 $$\begin{aligned} \left. |\psi _{8i}\right\rangle&={I_{ f_1^T}}\left. |\psi _7\right\rangle \nonumber \\&=\frac{-1}{8}(-3\left. |0000\right\rangle +\left. |0001\right\rangle +\left. |0010\right\rangle +\left. |0011\right\rangle +\left. |0100\right\rangle \nonumber \\&\quad +\left. |0101\right\rangle +\left. |0110\right\rangle +\left. |0111\right\rangle +\left. |1000\right\rangle +\left. |1001\right\rangle \nonumber \\&\quad +\left. |1010\right\rangle +\left. |1011\right\rangle +\left. |1100\right\rangle +\left. |1101\right\rangle +\left. |1110\right\rangle \nonumber \\&\quad -3\left. |1111\right\rangle )\otimes \left. |0\right\rangle \nonumber \\&\quad +\frac{1}{8}(-3\left. |0000\right\rangle +\left. |0001\right\rangle +\left. |0010\right\rangle +\left. |0011\right\rangle +\left. |0100\right\rangle \nonumber \\&\quad +\left. |0101\right\rangle +\left. |0110\right\rangle +\left. |0111\right\rangle +\left. |1000\right\rangle +\left. |1001\right\rangle \nonumber \\&\quad +\left. |1010\right\rangle +\left. |1011\right\rangle +\left. |1100\right\rangle +\left. |1101\right\rangle +\left. |1110\right\rangle \nonumber \\&\quad -3\left. |1111\right\rangle )\otimes \left. |1\right\rangle . \end{aligned}$$
    $\left\langle \alpha \right\rangle = \frac{{2*\frac{3}{8} + 6*\frac{{ - 1}}{8} + 8*\frac{{ - 1}}{8}}}{{16}} = \frac{{ - 1}}{{16}}$ for the amplitudes of states to the illustrative example which product to the state $\left. |0\right\rangle$. Then, the inversions about the mean of the amplitudes for these states are as follows:
    86 $$\begin{aligned} -\beta +2*\left\langle \alpha \right\rangle&=\frac{-3}{8}+2*\frac{-1}{16}=\frac{-1}{2}, \end{aligned}$$
    87 $$\begin{aligned} -\alpha +2*\left\langle \alpha \right\rangle&=\frac{1}{8}+2*\frac{-1}{16}=0, \end{aligned}$$
    88 $$\begin{aligned} -\gamma +2*\left\langle \alpha \right\rangle&=\frac{1}{8}+2*\frac{-1}{16}=0, \end{aligned}$$
    and $\left\langle \alpha \right\rangle = \frac{{2*\frac{{ - 3}}{8} + 6*\frac{1}{8} + 8*\frac{1}{8}}}{{16}} = \frac{1}{{16}}$ for the amplitudes of states to the illustrative example which product to the state $\left. |1\right\rangle$. Then, the inversions about the mean of the amplitudes for these states are as follows:
    89 $$\begin{aligned} -\beta +2*\left\langle \alpha \right\rangle&=\frac{3}{8}+2*\frac{1}{16}=\frac{1}{2}, \end{aligned}$$
    90 $$\begin{aligned} -\alpha +2*\left\langle \alpha \right\rangle&=\frac{-1}{8}+2*\frac{1}{16}=0, \end{aligned}$$
    91 $$\begin{aligned} -\gamma +2*\left\langle \alpha \right\rangle&=\frac{-1}{8}+2*\frac{1}{16}=0, \end{aligned}$$
    Hence, applying the Grover operator *G* can take this form:
    92 $$\begin{aligned} \left. |\psi _{8ii}\right\rangle = G \left. |\psi _{8i}\right\rangle =\frac{-1}{2}\left( \left. |0000\right\rangle +\left. |1111\right\rangle \right) \otimes \left. |0\right\rangle +\frac{1}{2}\left( \left. |0000\right\rangle +\left. |1111\right\rangle \right) \otimes \left. |1\right\rangle . \end{aligned}$$
  - Observe the system.
    The following result is obtained after applying $I_{ f_2^T}$, *G*, $I_{ f_1^T}$, *G* to the illustrative example.
    93 $$\begin{aligned} \left. |\psi _{8iii}\right\rangle&=\frac{-1}{4}(-\left. |0000\right\rangle +\left. |0001\right\rangle +\left. |0011\right\rangle +\left. |0101\right\rangle +\left. |0111\right\rangle \nonumber \\&\quad +\left. |1001\right\rangle +\left. |1011\right\rangle -\left. |1111\right\rangle )\otimes \left. |0\right\rangle \nonumber \\&\quad +\frac{1}{4}(-\left. |0000\right\rangle +\left. |0001\right\rangle +\left. |0011\right\rangle +\left. |0101\right\rangle +\left. |0111\right\rangle \nonumber \\&\quad +\left. |1001\right\rangle +\left. |1011\right\rangle - \left. |1111\right\rangle )\otimes \left. |1\right\rangle . \end{aligned}$$
3. Find the probability of a difference out of the *R* possible difference between ${\mathcal {N}}$ items as in Eq. (52).
  To the illustrative example, the difference that makes $f_{1}$ evaluates to true and $f_{2}$ evaluates to false can be obtained with the probability $= 0.125$ for $\left. |1\right\rangle ,\left. |3\right\rangle ,\left. |5\right\rangle ,\left. |7\right\rangle ,\left. |9\right\rangle , \left. |11\right\rangle .$

Arima algorithm with an adjustment can solve the illustrative example by searching for the truth assignment of $f_1$. Suppose that searching data $\left. |1\right\rangle ,\left. |3\right\rangle , \left. |5\right\rangle ,\left. |7\right\rangle , \left. |9\right\rangle$, and $\left. |11\right\rangle$ where $P = 2$.

The initial state described by $|\psi _{0}\rangle =\frac{1}{2\sqrt{2}}(1, 1, 0, 1, 0, 1, 0, 1, 0, 1, 0,1 ,0 ,0 ,0 ,1).$

$$|\psi _{1}\rangle = {I_{ f_1^T}}|\psi _{0}\rangle =\frac{1}{2\sqrt{2}}(1, -1, 0, -1, 0, -1, 0, -1, 0, -1, 0, -1, 0, 0, 0, 1).$$

$$|\psi _{2}\rangle = {G}|\psi _{1}\rangle =\frac{1}{4\sqrt{2}}(-3, 1, -1, 1, -1, 1, -1, 1, -1, 1, -1, 1, -1, -1, -1, -3)$$.

$$|\psi _{3}\rangle = {I_{ f_2^T}}|\psi _{2}\rangle =\frac{1}{4\sqrt{2}} (3, -1, -1, -1, -1, -1, -1, -1, -1, -1, -1, -1, -1, -1, -1, 3).$$

$$|\psi _{4}\rangle = {G}|\psi _{3}\rangle =\frac{1}{\sqrt{2}} (-1, 0, 0, 0, 0, 0, 0, 0, 0, 0, 0, 0, 0, 0, 0, -1).$$

$$|\psi _{5}\rangle = {I_{ f_1^T}}|\psi _{4}\rangle =\frac{1}{\sqrt{2}}(-1, 0, 0, 0, 0, 0, 0, 0, 0, 0, 0, 0, 0, 0, 0, -1)$$.

$$|\psi _{6}\rangle = {G}|\psi _{5}\rangle =\frac{1}{4\sqrt{2}} (3, -1, -1, -1, -1, -1, -1, -1, -1, -1, -1, -1, -1, -1,-1, 3).$$

$$|\psi _{7}\rangle = {I_{ f_2^T}}|\psi _{6}\rangle =\frac{1}{4\sqrt{2}}(-3, 1, -1, 1, -1, 1, -1, 1, -1, 1, -1, 1, -1, -1, -1, -3).$$

$$|\psi _{8}\rangle = {G}|\psi _{7}\rangle =\frac{1}{2\sqrt{2}}\ (1, -1, 0, -1, 0, -1, 0, -1, 0, -1, 0, -1, 0, 0, 0, 1).$$

Then the probability $(\frac{-1}{2\sqrt{2}})^2=0.125$ for any one of the following patterns: $\left. |1\right\rangle$, $\left. |3\right\rangle$, $\left. |5\right\rangle$, $\left. |7\right\rangle$, $\left. |9\right\rangle$ and $\left. |11\right\rangle$.

**Figure 3:**
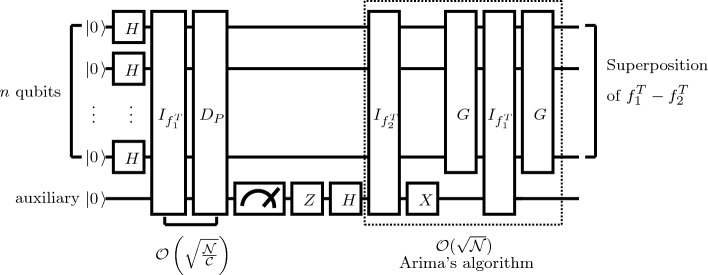
Quantum circuit for the proposed difference algorithm.

A pseudocode of difference operation is shown in Algorithm 3; the circuit of the proposed algorithm is shown in Fig. 3.

**Algorithm 3:**
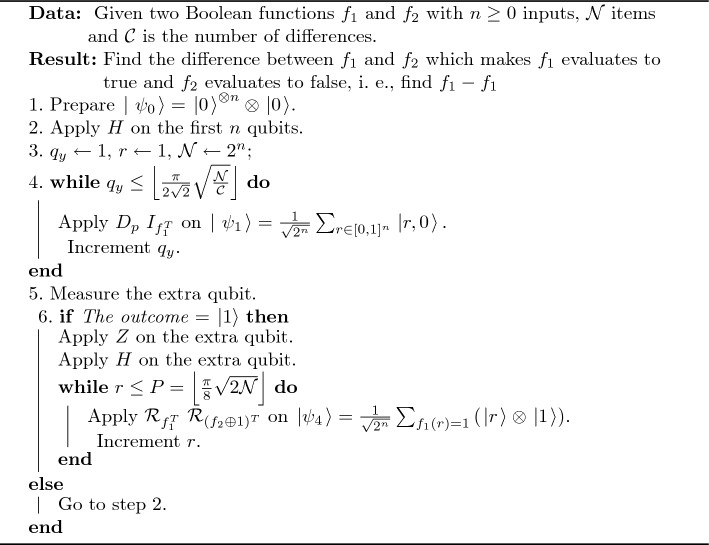
An Algorithm of Difference Operation.

### The proposed quantum algorithm for union operation

Given two Boolean functions $f_1$ and $f_2$, it is required to find the set of binary vectors that evaluates either $f_1$ or $f_2$ to True, i.e., to find the union between them. We need to find a partition of $f_1\cup f_2$, thus decomposing it into a complement of the intersection of distinct complement sets, i. e. , $f_1\cup f_2=\left( \left( f_1\oplus 1\right) \cap \left( f_2\oplus 1\right) \right) \oplus 1= f \oplus 1$ such that *f* is a Boolean function which represents all the truth value of the states of the system, and $f=\left( \left( f_1\oplus 1\right) \cap \left( f_2\oplus 1\right) \right)$. The steps of the proposed algorithm will be illustrated using the previous example:

1. Apply the algorithm in Sect. 3.2 to find the result of $$f=\left( f_1\oplus 1\right) \cap \left( f_2\oplus 1\right)$$ to the illustrative example.
  94 $$\begin{aligned} \left. |\psi \right\rangle&=\frac{-1}{2}\left( \left. |1101\right\rangle +\left. |1110\right\rangle \right) \otimes \left. |0\right\rangle +\frac{1}{2}\left( \left. |1101\right\rangle +\left. |1110\right\rangle \right) \otimes \left. |1\right\rangle . \end{aligned}$$
  As a result, *f* evaluates to true for each pattern in the set $$\{\left. {|13}\right\rangle {,}\left. { |14}\right\rangle \}$$.
2. Apply Younes et al. algorithm to find the result of $$f \oplus 1$$ as follows:
  - The preparation of the register consists of $$n+1$$ qubits, and all of them are in the state $$\left. |0\right\rangle$$. The auxiliary qubit is used to evaluate the Boolean function $$f_1$$. The state of the system is as in Eqs. (22) and (23).
  - (b). The initialization of the register in which the Hadamard gate *H* is applied on the first *n* qubits in parallel as in Eqs. (24) and (25).
  - This algorithm iterates the following steps for $$\left\lfloor \frac{\pi }{2\sqrt{2}}\sqrt{\frac{{\mathcal {N}}}{\mathcal C}}\right\rfloor .$$
    (i) Apply the oracle operator $$I_{ f}$$ on $$n+1$$ qubits where $$I_{ f}$$ evaluates the Boolean function *f* as follows:
    95 $$\begin{aligned} \left. \left| \psi _2\right. \right\rangle \ {}&=I_{ f}\left. \left| \psi _1\right. \right\rangle .\nonumber \\&=\frac{1}{\sqrt{2^n}}\ \sum _{r=0}^{2^n-1}{\ \left. \left| r\right. \right\rangle }\otimes \left. \left| f(r)\right. \right\rangle .\nonumber \\&=\frac{1}{\sqrt{2^n}}\sum _{ r\in {[0,1]^{n}}} \left. \left| r, f(r)\right. \right\rangle . \end{aligned}$$
    such that
    96 $$\begin{aligned} I_{ f}|r, 0\rangle =\left\{ \begin{array}{l} |r, 0\rangle ,\ \ \ f(r)=0 \\ |r, 1\rangle , \ \ \ f(r)=1 \end{array}\right. \end{aligned}$$
    When applying Eq. (95) to the illustrative example, the form will be as follows
    $$\begin{aligned} \left. |\psi _2\right\rangle&\nonumber =I_{f}\left. |\psi _1\right\rangle =I_{ f}\left( H^{\otimes 4}\otimes I\right) \left. |00000\right\rangle \\&\nonumber =\frac{1}{4}(\left. |00000\right\rangle +\left. |00010\right\rangle +\left. |00100\right\rangle +\left. |00110\right\rangle +\left. |01000\right\rangle \\&\nonumber \quad +\left. |01010\right\rangle +\left. |01100\right\rangle +\left. |01110\right\rangle +\left. |10000\right\rangle +\left. |10010\right\rangle \\&\nonumber \quad + \left. |10100\right\rangle +\left. |10110\right\rangle +\left. |11000\right\rangle +\left. |11011\right\rangle +\left. |11101\right\rangle \\ {}&\quad +\left. |11110\right\rangle ). \end{aligned}$$
    (ii) Apply *X* on the auxiliary qubit (Younes* and Miller, 2004) as follows:
    97 $$\begin{aligned} \left. \left| \psi _3\right. \right\rangle&=(I_n\otimes X)\otimes \left. \left| \psi _2\right. \right\rangle .\nonumber \\&=\frac{1}{\sqrt{2^n}}\sum ^{{{2}}^n-{ 1}}_{r{ =0}}(I_n\otimes X)\otimes \left. \left| r,f(r)\right. \right\rangle . \end{aligned}$$
    When applying Eq. (97) to the illustrative example, the form will be as follows
    98 $$\begin{aligned} \left. |\psi _3\right\rangle&=\left( I^{\otimes 4}\otimes X\right) \left. |\psi _2\right\rangle \nonumber \\&=\frac{1}{4}(\left. |00001\right\rangle +\left. |00011\right\rangle +\left. |00101\right\rangle +\left. |00111\right\rangle +\left. |01001\right\rangle \nonumber \\&\quad +\left. |01011\right\rangle +\left. |01101\right\rangle +\left. |01111\right\rangle +\left. |10001\right\rangle +\left. |10011\right\rangle \nonumber \\&\quad + \left. |10101\right\rangle +\left. |10111\right\rangle +\left. |11001\right\rangle +\left. |11010\right\rangle +\left. |11100\right\rangle \nonumber \\&\quad +\left. |11111\right\rangle ). \end{aligned}$$
    (iii) After that, the algorithm applies the partial diffusion $$D_p$$ on the $$n+1$$ qubits as follows:
    99 $$\begin{aligned} \left. \left| \psi _4\right. \right\rangle&=D_p\ \left. \left| \psi _3\right. \right\rangle \nonumber \\&=a_q\sum _{r=0}^{2^n-1}{' (}\left. \left| r\right. \right\rangle \otimes \ \left. \left| 0\right. \right\rangle )+b_q\sum _{r=0}^{2^n-1}{''\ (}\left. \left| r\right. \right\rangle \otimes \ \left. \left| 0\right. \right\rangle )\nonumber \\&\quad + c_q\sum _{r=0}^{2^n-1}{''\ (}\left. \left| r\right. \right\rangle \otimes \ \left. \left| 1\right. \right\rangle ). \end{aligned}$$
    The mean of amplitudes to the illustrative example is
    100 $$\begin{aligned} \left\langle \alpha \right\rangle =\frac{\mathcal N-m}{N\sqrt{{\mathcal {N}}}}=\frac{1}{32}, \end{aligned}$$
    then the inversions about the mean of the amplitudes are
    101 $$\begin{aligned} a_q=2*\left\langle \alpha \right\rangle -\frac{1}{\sqrt{{\mathcal {N}}}}\ =\frac{-3}{16},\ b_q{=}2*\left\langle \alpha \right\rangle =\frac{1}{16},\ c_q{=}-\frac{{ 1}}{\sqrt{\mathcal N}}=\frac{-1}{4} \end{aligned}$$.
    Hence, applying the partial diffusion $$D_p$$ can take this form
    102 $$\begin{aligned} \left. |\psi _4\right\rangle&={D_p}\left. |\psi _3\right\rangle \nonumber \\&= \frac{1}{16}(\left. |0000\right\rangle +\left. |0001\right\rangle +\left. |0010\right\rangle +\left. |0011\right\rangle +\left. |0100\right\rangle \nonumber \\&\quad +\left. |0101\right\rangle +\left. |0110\right\rangle +\left. |0111\right\rangle +\left. |1000\right\rangle +\left. |1001\right\rangle 
\nonumber \\&\quad +\left. |1010\right\rangle +\left. |1011\right\rangle +\left. |1100\right\rangle -3\left. |1101\right\rangle -3\left. |1110\right\rangle \nonumber \\&\quad +\left. |1111\right\rangle )\otimes \left. |0\right\rangle \nonumber \\&\quad -\frac{1}{4}(\left. |0000\right\rangle +\left. |0001\right\rangle +\left. |0010\right\rangle +\left. |0011\right\rangle +\left. |0100\right\rangle \nonumber \\&\quad +\left. |0101\right\rangle +\left. |0110\right\rangle +\left. |0111\right\rangle +\left. |1000\right\rangle +\left. |1001\right\rangle \nonumber \\&\quad +\left. |1010\right\rangle +\left. |1011\right\rangle +\left. |1100\right\rangle +\left. |1111\right\rangle )\otimes \left. |1\right\rangle . \end{aligned}$$
  - (d). Apply the measurement on the auxiliary qubit. The probability to get $$\left. \left| 1\right. \right\rangle$$ on the auxiliary qubit is $${\mathcal {C}}|c_q|^2$$ and the superposition can be represented as follows:
    103 $$\begin{aligned} \left. \left| \psi _5\right. \right\rangle =\frac{1}{\sqrt{2^n}}\sum _ {f(r)=0}{(\left. \left| r\right. \right\rangle \otimes \left. \left| 1\right. \right\rangle ).} \end{aligned}$$
    Applying the measurement on the auxiliary qubit to the illustrative example is as follows:
    104 $$\begin{aligned} \left. |\psi _{5}\right\rangle&\nonumber =\frac{-1}{\sqrt{14}}(\left. |0000\right\rangle +\left. |0001\right\rangle +\left. |0010\right\rangle +\left. |0011\right\rangle +\left. |0100\right\rangle \\ {}&\nonumber \quad +\left. |0101\right\rangle +\left. |0110\right\rangle +\left. |0111\right\rangle +\left. |1000\right\rangle +\left. |1001\right\rangle \\ {}&\quad +\left. |1010\right\rangle +\left. |1011\right\rangle +\left. |1100\right\rangle +\left. |1111\right\rangle )\otimes \left. |1\right\rangle \end{aligned}$$
    To the illustrative example, the union that makes $$f_{{1}}$$ and $$f_{{ 2}}$$ evaluate to True can be obtained with the probability $${(\frac{-1}{\sqrt{14}})}^2=0.071$$ for $$\left. |0\right\rangle$$, $$\left. |1\right\rangle$$, $$\left. |2\right\rangle$$, $$\left. |3\right\rangle$$, $$\left. |4\right\rangle$$, $$\left. |5\right\rangle$$, $$\left. |6\right\rangle$$, $$\left. |7\right\rangle$$, $$\left. |8\right\rangle$$, $$\left. |9\right\rangle$$, $$\left. |10\right\rangle$$, $$\left. |11\right\rangle$$, $$\left. |12\right\rangle$$, or $$\left. |15\right\rangle$$.

**Figure 4:**
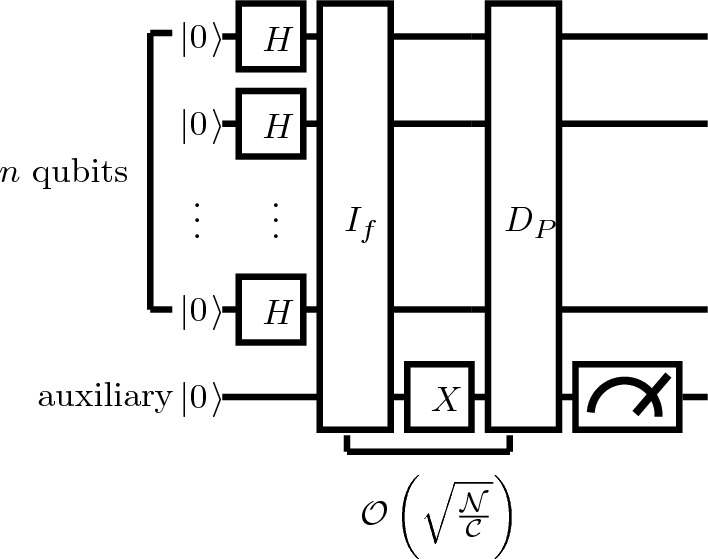
Quantum circuit to find the result of $f \oplus 1$.

A pseudocode of union operation is shown in Algorithm 4; The circuit of the proposed algorithm is shown in Figs. 2 and 4.

**Algorithm 4:**
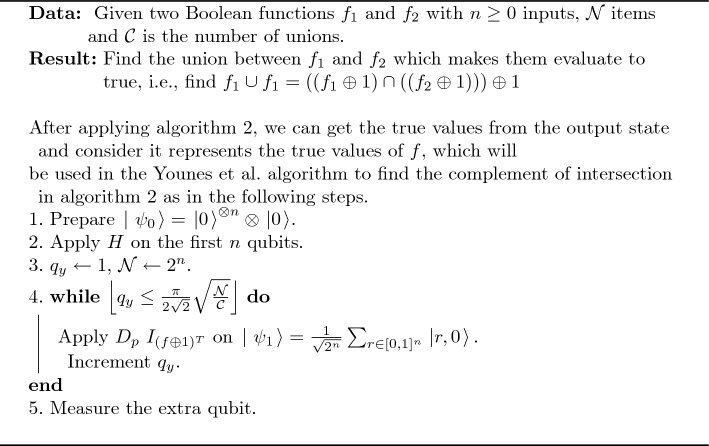
An Algorithm of Union Operation.

## Analysis of the proposed algorithms

### Dynamics of the proposed algorithms

Assume that *R* represents the number of possible solutions, $\alpha _i(T)$ represents the amplitudes of desired (searching) of the stored data at a time step *T* such that $1\le i\le R$, $\beta _j(T)$ represents the amplitudes of non-solutions of the stored data such that $R+1\le j\le m$, $\gamma _k(T)$ represents the amplitudes of other data (which were not in the prepared superposition) such that $m\le k\le {\mathcal {N}}$, $\overline{\alpha }(T)$ represents the average of the amplitudes of desired (searching) of the stored data, $\overline{\beta }(T)$ represents the average of the amplitudes of non-solutions of the stored data, $\overline{\gamma }(T)$ represents the average of the amplitudes of other data (which were not in the prepared superposition). Then, we can define the form of the searching phase $\acute{\left. |\psi \right\rangle }$ as follows:

105 $$\begin{aligned} \acute{\left. |\psi \right\rangle }&=\sum ^{\mathcal N-1}_{r=0}\left( \frac{1}{R}\sum ^R_{i=1}{\alpha _i(T)}\left( \left. |r\right\rangle \otimes \left. |1\right\rangle \right) +\frac{1}{m-R}\sum ^m_{j=R+1}{\beta _j(T)}\left( \left. |r\right\rangle \otimes \left. |0\right\rangle \right) \right. \nonumber \\&\quad \left. +\frac{1}{\mathcal N-m}\sum ^{\mathcal {N}}_{k=m+1}{\gamma _k(T)}\left( \left. |r\right\rangle \otimes \left. |1\right\rangle \right) \right) \nonumber \\&=\sum ^{\mathcal N-1}_{r=0}{\left( \overline{\alpha }\left( T\right) \left( \left. |r\right\rangle \otimes \left. |1\right\rangle \right) +\overline{\beta }\left( T\right) \left( \left. |r\right\rangle \otimes \left. |0\right\rangle \right) +\overline{\gamma }\left( T\right) \left( \left. |r\right\rangle \otimes \left. |1\right\rangle \right) \right) }, \end{aligned}$$

and the average of the amplitudes are

106 $$\begin{aligned} \overline{{\alpha }}\left( T\right)= & {} \frac{1}{{ R}}\sum ^{{ R}}_{i=1}{\alpha _i\left( T\right) .} \end{aligned}$$

107 $$\begin{aligned} \overline{{\beta }}\left( T\right)= & {} \frac{1}{{ m}-{ R}}\sum ^{{ m}}_{j={ R}+1}{\beta _j\left( T\right) .} \end{aligned}$$

108 $$\begin{aligned} \overline{{\gamma }}\left( T\right)= & {} \frac{1}{{\mathcal {N}}-{ m}}\sum ^{\mathcal {N}}_{{ k}={ m}+1}{\gamma _k\left( T\right) .} \end{aligned}$$

Let *W*(*T*) be the weighted averages over states

109 $$\begin{aligned} W\left( T\right)&=\frac{-\sum ^R_{i=1}{\alpha _i\left( T\right) +\sum ^m_{j=R+1}{\beta _j\left( T\right) }+\sum ^\mathcal N_{k=m+1}{\gamma _k\left( T\right) }}}{{\mathcal {N}}}\nonumber \\&=\frac{(\mathcal N-m)\overline{\gamma }\left( T\right) +(m-R)\overline{\beta }\left( T\right) -(R)\overline{\gamma }\left( T\right) }{\mathcal N}. \end{aligned}$$

After that, using Biron results^43^, the following relation holds:

110 $$\begin{aligned} \overline{{\alpha }}\left( T+1\right)= & {} 2\ w\left( \acute{T}\right) +\overline{{\alpha }}\left( \acute{T}\right) \end{aligned}$$

111 $$\begin{aligned} \overline{{\beta }}\left( T+1\right)= & {} 2\ w\left( \acute{T}\right) +\overline{{\beta }}\left( \acute{T}\right) \end{aligned}$$

112 $$\begin{aligned} \overline{{\gamma }}\left( T+1\right)= & {} 2\ w\left( \acute{T}\right) -\overline{{\gamma }}\left( \acute{T}\right) \end{aligned}$$

By solving Eqs. (110), (111) and (112), we obtained the following equation:

113 $$\begin{aligned} \left[ \begin{array}{c} \overline{\alpha }\left( T+1\right) \\ \overline{\beta }\left( T+1\right) \\ \overline{\gamma }\left( T+1\right) \end{array} \right] =\left[ \begin{array}{ccc} \frac{{\mathcal {N}}^2-8{\mathcal {N}}+8(m-R)}{{\mathcal {N}}^2} &{} \frac{8({\mathcal {N}}-m)(m-R)}{{\mathcal {N}}^2} &{} \frac{4({\mathcal {N}}-m)({\mathcal {N}}-2m-2R+1)}{{\mathcal {N}}^2} \\ \frac{-8({\mathcal {N}}-(m-R))}{{\mathcal {N}}^2} &{} \frac{{8({\mathcal {N}}-m)(m-R)-{\mathcal {N}}}^2}{N^2} &{} \frac{4({\mathcal {N}}-m)({\mathcal {N}}-2m-2R+1)}{{\mathcal {N}}^2} \\ \frac{-4({\mathcal {N}}-2(m-R))}{{\mathcal {N}}^2} &{} \frac{4({\mathcal {N}}-2m)(m-R)}{{\mathcal {N}}^2} &{} \frac{-8m({\mathcal {N}}-m-R+1)+{\mathcal {N}}^2}{{\mathcal {N}}^2} \end{array} \right] *\left[ \begin{array}{c} \overline{\alpha }\left( T\right) \\ \overline{\beta }\left( T\right) \\ \overline{\gamma }\left( T\right) \end{array} \right] \end{aligned}$$

Suppose that the system $\left. \left| {{\psi }}_{{9}}\right. \right\rangle =\alpha _{i}(\left. \left| { r}\right. \right\rangle \otimes \left. \left| 1\right. \right\rangle )+\beta _{i}(\left. \left| { r}\right. \right\rangle \otimes \left. \left| { 0}\right. \right\rangle )$ and to find the probability of a match out of the *R* possible match between ${\mathcal {N}}$ items as follows

After the first iteration $(P\ =\ 1)$, then the probabilities of the system according to the proposed quantum algorithms will be as follows:

1. The probability $P_r^{(1)}$ to find a match out of the *R* possible match is calculated as follows
  114 $$\begin{aligned} {P_r}^{(1)}={2*\left( {\alpha }_2\right) }^2, \end{aligned}$$
  such that $$\left\langle {\alpha }_1\right\rangle =\frac{m-2R}{\mathcal {N}\sqrt{\mathcal {N}}},\ {\beta }_1=\frac{-1}{\sqrt{\mathcal {N}}}+2*\left\langle {\alpha }_1\right\rangle ,\ {\alpha }_1=\frac{1}{\sqrt{\mathcal {N}}}+2*\left\langle {\alpha }_1\right\rangle ,\ {\gamma }_1=2*\left\langle {\alpha }_1\right\rangle ,\ \left\langle {\alpha }_2\right\rangle =\frac{-1*\left( {\beta }_1\left( m-R\right) +{\alpha }_1R+{\gamma }_1\left( \mathcal {N}-m\right) \right) }{\mathcal {N}},\ {\alpha }_2=-{\alpha }_1+2*\left\langle {\alpha }_2\right\rangle ,\ {\beta }_2=-{\beta }_1+2*\left\langle {\alpha }_2\right\rangle ,\ {\gamma }_2={\gamma }_1+2*\left\langle {\alpha }_2\right\rangle .$$
2. The probability $P_s^{(1)}$ to find undesired results out of the stored states is given by:
  115 $$\begin{aligned} P_s^{(1)}={2*\left( {\beta }_2\right) }^2. \end{aligned}$$
3. The probability $P_e^{(1)}$ to find undesired results out of the unstored states is given by:
  116 $$\begin{aligned} P_e^{(1)}={2*\left( {\gamma }_2\right) }^2. \end{aligned}$$

Notice that

117 $$\begin{aligned} RP_r^{(1)}+(m-R)P_s^{(1)}+(\mathcal {N}-m)P_e^{(1)}=1, \end{aligned}$$

moreover, the probabilities of the system after the second iteration $(P\ =\ 2)$ will be as follows:

1. The probability $P_r^{(2)}$ to find a match out of the *R* possible match is calculated as follows
  118 $$\begin{aligned} P_r^{(2)}={2*\left( {\alpha }_4\right) }^2, \end{aligned}$$
  such that $$\left\langle {\alpha }_3\right\rangle =\frac{{\beta }_2(m-R)-{\alpha }_2R}{\mathcal {N}},\ {\alpha }_3={\alpha }_2+2*\left\langle {\alpha }_3\right\rangle ,\ {\beta }_3=-{\beta }_2+2*\left\langle {\alpha }_3\right\rangle ,\ {\gamma }_3={\gamma }_2+2*\left\langle {\alpha }_3\right\rangle ,\ \left\langle {\alpha }_4\right\rangle =\frac{-1*\left( {\beta }_3\left( m-R\right) +{\alpha }_3R-{\gamma }_3\left( \mathcal {N}-m\right) \right) }{\mathcal {N}},\ {\alpha }_4={\alpha }_3+2*\left\langle {\alpha }_4\right\rangle ,\ {\beta }_4={\beta }_3+2*\left\langle {\alpha }_4\right\rangle ,\ {\gamma }_4=-{\gamma }_3+2*\left\langle {\alpha }_4\right\rangle .$$
2. The probability $P_s^{(2)}$ to find undesired results out of the stored states is given by:
  119 $$\begin{aligned} P_s^{(2)}={2*\left( {\beta }_4\right) }^2. \end{aligned}$$
3. The probability $P_e^{(2)}$ to find undesired results out of the unstored states is given by:
  120 $$\begin{aligned} P_e^{(2)}={2*\left( {\gamma }_4\right) }^2. \end{aligned}$$

In general, the probabilities of the system after $(P\ \ge \ 2)$ iterations will be as follows:

1. The probability $P_r^{(P)}$ to find a match out of the *R* possible match is calculated as follows
  121 $$\begin{aligned} P_r^{(P)}={2*\left( {\alpha }_{P+2}\right) }^2, \end{aligned}$$
  such that $$\left\langle {\alpha }_{P+1}\right\rangle =\frac{{\beta }_P(m-R)-{\alpha }_PR}{\mathcal {N}},\ {\alpha }_{P+1}={\alpha }_P+2*\left\langle {\alpha }_{P+1}\right\rangle ,\ {\beta }_{P+1}=-{\beta }_P+2*\left\langle {\alpha }_{P+1}\right\rangle ,\ {\gamma }_{P+1}={\gamma }_P+2*\left\langle {\alpha }_{P+1}\right\rangle ,\ \left\langle {\alpha }_{P+2}\right\rangle =\frac{-1*\left( {\beta }_{P+1}\left( m-R\right) +{\alpha }_{P+1}R-{\gamma }_{P+1}\left( \mathcal {N}-m\right) \right) }{\mathcal {N}},\ {\alpha }_{P+2}={\alpha }_{P+1}+2*\left\langle {\alpha }_{P+2}\right\rangle ,\ {\beta }_{P+2}={\beta }_{P+1}+2*\left\langle {\alpha }_{P+2}\right\rangle ,\ {\gamma }_{P+2}=-{\gamma }_{P+1}+2*\left\langle {\alpha }_{P+2}\right\rangle .$$
2. The probability $P_s^{(P)}$ to find undesired results out of the stored states is given by:
  122 $$\begin{aligned} P_s^{(P)}={2*\left( {\beta }_{P+2}\right) }^2. \end{aligned}$$
3. The probability $P_e^{(P)}$ to find undesired results out of the unstored states is given by:
  123 $$\begin{aligned} P_e^{(P)}={2*\left( {\gamma }_{P+2}\right) }^2. \end{aligned}$$

The number of iterations must be an integer,

124 $$\begin{aligned} P \cong \frac{\pi }{2\sqrt{2}}\sqrt{\frac{\mathcal {N}}{\mathcal C}}+\frac{\pi }{8}\sqrt{2\mathcal {N}}=\mathcal {O}\left( \sqrt{\mathcal {N}}\right) \ \ or\ \ P \cong \frac{\pi }{2\sqrt{2}}\sqrt{\frac{\mathcal {N}}{\mathcal C}}+\frac{\pi }{8}\sqrt{\mathcal {N}}=\mathcal {O}\left( \sqrt{\mathcal {N}}\right) \end{aligned}$$

Quantifying the quantum resources needed to implement the proposed algorithms is crucial for evaluating their feasibility and practicality. The first stage of the proposed algorithms, which utilizes the Younes et al. algorithm, operates on $n+1$ qubits. In the second stage, a modified version of the Arima algorithm is employed, operating on *n* qubits. Overall, the total number of qubits is $\mathcal {O}(n)$.

### Comparative analysis

Comparing the proposed algorithms with relevant algorithms in literature, the proposed intersection algorithm runs in $\mathcal {O}\left( \sqrt{\mathcal {N}}\right)$, whereas Pang et al.^27^ runs in $\mathcal {O}\left( \sqrt{\left| A\right| \times \left| B\right| \times \left| I\right| }\right)$ for set operation $I=A\cap B$ such that | | denotes the size of the set; moreover, Pang et al. algorithm finds only the true intersection, whereas the proposed algorithms can be used for true and false intersection.

Comparing the proposed algorithms with K. El-Wazan^28^, the proposed quantum algorithms find the true intersection, false intersection, difference, and union between any two Boolean functions in $\mathcal {O}\left( \sqrt{\mathcal {N}}\right)$, whereas K. El-Wazan algorithm finds only common matches between databases in $\mathcal {O}\left( \sqrt{\mathcal {M}/\mathcal {C}}\right)$, such that $\mathcal {M}$ is the number of records for each database and $\mathcal {C}$ are the common entries between those databases.

Comparing the proposed algorithm for intersection with Jóczik et al.^29^, both algorithms run in $\mathcal {O}\left( \sqrt{\mathcal {N}}\right)$. The proposed algorithm can find arbitrary intersection while S. Jóczik et al. algorithm can find the intersection of two sets *A* and *B* when the size of the intersection is in the power set of *A*; moreover, Jóczik et al. cannot find the false intersection between these two sets.

## Discussion

Set operations such as intersection, difference, and union have many applications such as database query optimization, signal processing^27^, cryptography, collision problem^30^, quantum image processing^44^, and quantum machine learning^45^.

This study proposed efficient quantum algorithms for performing set operations on Boolean functions. Specifically, the algorithms leverage quantum principles such as amplitude amplification to achieve quadratic speedups over classical techniques. A key advantage is the $\mathcal {O}\left( \sqrt{\mathcal {N}}\right)$ runtime scaling, outperforming classical approaches. This improved scaling suggests a potential practical impact for domains that motivate this work, such as database optimization, cryptography, and machine learning.

Performing set operations on Boolean functions is of special importance, given their significant applications in domains such as databases, cryptography, and machine learning. In database systems, intersections are utilized to merge tables, while finding differences between functions is important in query optimization to help in eliminating unwanted records. Boolean operations can be used to secure computation between two-parties , enabling private function evaluation. Moreover, in machine learning, the identification of relationships between feature sets representing classes or categories is essential for classification algorithms^24,25^. The development of specialized quantum algorithms for these set operations holds immense potential for achieving exponential speedups by harnessing the benefits of quantum parallelism. This has the potential to greatly enhance the scalability of applications dealing with large datasets, particularly those processing amount of data. Hash functions are used in different applications, for examples cryptographic protocols, so a collision for a function is of particular interest in cryptology^30^. These protocols depend on finding collisions in such functions. The problem is to find so-called claws in pairs of functions; the proposed quantum algorithms in this paper can be used to solve the problem of finding collisions using the intersection algorithm and solve claws in pairs of functions using the difference algorithm. The hash function is used in the cryptographic protocols to test the Boolean function’s probability that requires set operations.

## Conclusion

In this paper, four quantum algorithms for set operations on two Boolean functions $f_1$, $f_2$ with *n* inputs are presented. The proposed Algorithm 1 finds the set of binary vectors that evaluates both $f_1$ and $f_2$ to True, i.e. it finds the true intersection between them. Algorithm 2 finds the set of binary vectors that evaluates both $f_1\oplus 1$ and $f_{2 }\oplus 1$ to True, i.e. it finds the False intersection between them. Algorithm 3 finds the set of binary vectors that evaluates $f_1$ to True and evaluates $f_2$ to False, i.e. it finds the difference between $f_1$ and $f_2$. Furthermore, Algorithm 4 finds the set of binary vectors that evaluates either $f_1$ or $f_2$ to True, i.e. it finds the union between them.

The proposed algorithms use amplitude amplification techniques and handle the non-uniform amplitudes’ distribution case for the system with multi-solutions. All algorithms are based on two algorithms; Younes et al. algorithm is used in the preparation step and an amended version of Arima’s algorithm is used in the searching step. Younes et al. algorithm prepares an incomplete superposition of the truth set of the first Boolean function $f_1$ using an amplitude amplification technique that employs entanglement and partial diffusion, while Arima’s algorithm is used with an oracle that represents the second Boolean function $f_2$ to search for a solution that represents the result of the set operator using phase shift inversion about the mean.

The proposed quantum algorithms can be used to apply set operations on two arbitrary Boolean functions in $\mathcal {O}\left( \sqrt{\mathcal {N}}\right)$ to find the solutions with high probability. The algorithms also demonstrated flexibility to compute arbitrary set relations compared to previous approaches. Resource estimates indicated polynomial number of qubits, suggesting potential near-term feasibility. Overall, the work developed practical quantum techniques advancing the capability to solve important set transformation problems. Future research includes optimizing resource costs, experiment implementation the algorithms, and customizing the approach to be used in different domains such as database queries, cryptography and machine learning. Furthermore, in the future, the proposed algorithms can be extended to perform set operations on more than two Boolean functions.

## Data Availability

All data are included in the paper.

## Code availability

Code is available on demand.
